# Artificial Intelligence of Things for Next-Generation Predictive Maintenance

**DOI:** 10.3390/s25247636

**Published:** 2025-12-16

**Authors:** Taimia Bitam, Aya Yahiaoui, Djallel Eddine Boubiche, Rafael Martínez-Peláez, Homero Toral-Cruz, Pablo Velarde-Alvarado

**Affiliations:** 1 LEREESI Laboratory, HNS-RE2SD, Batna 05000, Algeria; 2 Unidad Académica de Computación, Universidad Politécnica de Sinaloa, Mazatlán 82199, Mexico; 3 Departamento de Ingeniería de Sistemas y Computación, Universidad Católica del Norte, Antofagasta 1270709, Chile; 4 Departamento de Ingeniería y Tecnología, Universidad Autónoma del Estado de Quintana Roo, Chetumal 77019, Mexico; 5 Unidad Académica de Ciencias Básicas e Ingenierías, Universidad Autónoma de Nayarit, Tepic 63000, Mexico; pvelarde@uan.edu.mx

**Keywords:** Artificial Intelligence of Things, predictive maintenance, smart manufacturing, Industry 5.0

## Abstract

Industry 5.0 introduces a shift toward human-centric, sustainable, and resilient industrial ecosystems, emphasizing intelligent automation, collaboration, and adaptive operations. Predictive Maintenance (PdM) plays a critical role in this transition, addressing the limitations of traditional maintenance approaches in increasingly complex and data-driven environments. The convergence of Artificial Intelligence and the Industrial Internet of Things, referred to as the Artificial Intelligence of Things (AIoT), enables real-time sensing, learning, and decision-making for advanced fault detection, Remaining Useful Life estimation, and prescriptive maintenance actions. This study provides a systematic and structured review of AIoT-enabled PdM aligned with Industry 5.0 objectives. It presents a unified taxonomy integrating AI models, Industrial Internet of Things (IIoT) infrastructures, and AIoT architectures; reviews AI-driven techniques, sector-specific implementations in manufacturing, transportation, and energy; and analyzes emerging paradigms such as Edge–Cloud collaboration, federated learning, self-supervised learning, and digital twins for autonomous and privacy-preserving maintenance. Furthermore, this paper synthesizes strengths, limitations, and cross-industry challenges, and outlines future research directions centered on explainability, data quality and heterogeneity, resource-constrained intelligence, cybersecurity, and human–AI collaboration. By bridging technological advancements with Industry 5.0 principles, this review contributes a comprehensive foundation for the development of scalable, trustworthy, and next-generation AIoT-based predictive maintenance systems.

## 1. Introduction

In recent years, industrial operations have increasingly been shaped by the integration of automation, artificial intelligence (AI), and data analytics, leading to smarter and more connected systems. While Industry 4.0 emphasized cyber-physical systems, digital twins, and end-to-end connectivity, Industry 5.0 advances these concepts by focusing on human-centricity, environmental sustainability, and systemic resilience. Instead of prioritizing only efficiency and automation, Industry 5.0 promotes closer collaboration between human expertise and intelligent technologies to create adaptive, personalized, and sustainable production ecosystems.

As industrial systems become more complex and dynamic, maintaining their operational efficiency, reliability, and longevity presents growing challenges. Traditional maintenance methods, such as reactive maintenance, which addresses failures post-occurrence, and preventive maintenance, which relies on fixed schedules, often result in unnecessary interventions, increased costs, and unplanned downtime. These inefficiencies have driven the transition toward Predictive Maintenance (PdM), a data-driven approach that leverages sensor data, AI models, and analytics to detect early indicators of equipment degradation. PdM minimizes disruptions, reduces maintenance costs, and extends asset life, thereby enhancing productivity and system resilience.

The emergence of the Artificial Intelligence of Things (AIoT), the integration of AI capabilities with IoT infrastructures, has further elevated the potential of PdM. In AIoT-enabled systems, distributed sensors continuously collect operational data (e.g., temperature, vibration, pressure), while AI algorithms process these data streams in real time to detect anomalies, predict faults, and recommend actions. This synergy enables intelligent, autonomous, and scalable maintenance frameworks that align with Industry 5.0 values. Edge computing, federated learning, and hybrid AI models further enhance responsiveness, privacy, and system adaptability, making AIoT a cornerstone for future-ready maintenance strategies.

This survey examines the role of AIoT in facilitating PdM within Industry 5.0. It provides a detailed overview of foundational concepts, explores sector-specific implementations, and presents a structured taxonomy of AIoT-powered techniques. This study also analyzes key architectural trends, evaluates performance trade-offs, and highlights critical challenges in deployment and scalability. By synthesizing current research and identifying future opportunities, this survey aims to guide the development of intelligent, resilient, and human-aligned maintenance solutions in next-generation industrial contexts.

### Contributions of This Survey

This survey offers several key contributions to the field of AIoT-driven predictive maintenance in the context of Industry 5.0:

- C1: Industry 5.0 and AIoT Foundations. Provides a comprehensive overview of the Industry 5.0 paradigm, the evolution of maintenance strategies, and the strategic role of AIoT as the convergent enabler of next-generation predictive maintenance.
- C2: Unified AIoT Taxonomy and Background. Introduces a structured taxonomy integrating Artificial Intelligence (AI), the Industrial Internet of Things (IIoT), and their convergence into the AIoT framework, covering core technologies, communication protocols, and architectural components.
- C3: Systematic Review of AI and IIoT Techniques. Presents a structured analysis of AI techniques (fault detection, Remaining Useful Life (RUL) prediction, maintenance scheduling) and IIoT implementations across key industrial sectors (manufacturing, transportation, energy).
- C4: Analysis of AIoT-Enhanced Maintenance Paradigms. Synthesizes how AIoT enhances established maintenance methodologies, including Condition-Based Maintenance (CBM), Prognostics and Health Management (PHM), Prescriptive Maintenance, and hybrid AIoT strategies.
- C5: Review of Next-Generation AIoT Architectures. Examines emerging AIoT system paradigms that enable scalable and intelligent PdM, including Edge–Cloud Collaboration, Federated and Self-Adaptive Learning, and Digital Twin-driven maintenance.
- C6: Critical Synthesis and Future Directions. Highlights open challenges, limitations, and concrete future research directions related to explainability, data quality, resource constraints, cybersecurity, and human–AI collaboration in AIoT systems.

To facilitate traceability, Table 1 provides a clear mapping between each contribution, the corresponding manuscript sections, and the primary thematic focus.

## 2. Methodology

PRISMA Compliance Statement: This systematic review was conducted and reported in accordance with the PRISMA 2020 guidelines to ensure scientific rigor, transparency, and reproducibility (see Table S1 in the Supplementary Materials). The review protocol was not registered in a public registry. The PRISMA framework provides a structured and traceable procedure for identifying, screening, selecting, and including relevant studies across the interdisciplinary domains of AI, IIoT, and predictive maintenance in Industry 5.0 contexts.

### 2.1. Research Questions

This survey was developed around the following research questions (RQs), which shaped the search strategy, screening process, and thematic synthesis:

- RQ1: What are the recent advancements in AIoT-based predictive maintenance techniques aligned with the principles of Industry 5.0?
- RQ2: How are artificial intelligence and IIoT technologies integrated to support predictive maintenance across different industrial sectors?
- RQ3: What challenges, limitations, and future directions are identified in current literature concerning AIoT-based predictive maintenance systems?

### 2.2. Search Strategy and Data Sources

An extensive literature search was conducted across four major academic databases: Google Scholar, IEEE Xplore, Scopus, and Web of Science. This selection ensured broad disciplinary coverage, ranging from computer science and AI to industrial engineering and cyber-physical systems.

The search covered publications from January 2019 to September 2025, with the last update on 15 September 2025. To guarantee reproducibility, the exact Boolean search query used across databases is now provided below:

*(“Predictive Maintenance” OR “PdM” OR “Condition-based Maintenance” OR “CBM” OR “Prognostics and Health Management” OR “PHM” OR “Remaining Useful Life” OR “RUL”) AND (“Artificial Intelligence” OR “AI” OR “Machine Learning” OR “Deep Learning” OR “Transformer” OR “Federated Learning” OR “Reinforcement Learning”) AND (“Internet of Things” OR “IoT” OR “Industrial IoT” OR “IIoT” OR “AIoT” OR “Edge Computing” OR “Fog Computing” OR “Cloud Computing”)*

Additional domain-specific modifiers (e.g., “manufacturing”, “transportation”, “energy”, “railway”, “aerospace”) were applied during secondary filtering.

Inclusion of Web Sources: In addition to peer-reviewed literature, four high-authority web sources were consulted (European Commission Industry 5.0 reports, IEEE Spectrum articles, industrial white papers from recognized consortia, and trusted technology blogs with institutional authorship). These were included solely to provide contextual background on Industry 5.0 policy frameworks, emerging industrial standards, and real-world adoption trends—areas where academic publishing often lags. These sources were carefully selected based on authorship credibility, institutional affiliation, and citation in reputable academic works, and were not used as primary evidence for technical AIoT or PdM claims. They are cited only in introductory and background sections to frame the Industry 5.0 context.

### 2.3. Deduplication Procedure

All retrieved records were exported in RIS/BibTeX formats and processed through a two-step deduplication pipeline:

1. Automated deduplication: DOI matching, title similarity scoring, and metadata alignment using Zotero and a custom Python 3.11 script.
2. Manual verification: Inspection of borderline cases such as:
  - Preprint–journal duplicates;
  - Extended versions of conference papers;
  - Inconsistent author spellings or title formatting.

This process removed redundant entries before the screening phase.

### 2.4. Inclusion and Exclusion Criteria

To operationalize the review process, we defined a set of explicit, actionable inclusion and exclusion criteria.

#### 2.4.1. Inclusion Criteria

Studies were included if they met all of the following criteria:

- Written in English and published between 2019 and 2025.
- Address predictive maintenance (FDD, CBM, PHM, or RUL) using AI, IIoT, or integrated AIoT technologies.
- Provide empirical evidence (e.g., experiments, benchmarks, real-world or simulated datasets) or a clearly defined architecture enabling implementation.
- Contribute to at least one Industry 5.0 value: human-centricity, resilience, or sustainability.

#### 2.4.2. Exclusion Criteria

Studies were excluded if they met any of the following criteria:

- Duplicate entries across multiple databases.
- Non-English, non-peer-reviewed, or grey literature (except four validated contextual web sources).
- Works focusing solely on:
  – Non-maintenance industrial automation;
  – Unrelated IoT applications (e.g., healthcare, smart homes);
  – AI models without application to PdM contexts.

#### 2.4.3. Edge Cases

We explicitly documented and justified the handling of borderline or ambiguous studies:

- Conceptual frameworks without experiments were included only if they proposed implementable PdM architectures.
- Works related to safety, cybersecurity, or anomaly detection were included when directly linked to machine degradation, fault progression, or maintenance scheduling.
- General IoT/AI works were excluded unless they provided explicit PdM use cases.

### 2.5. Screening and Selection Process

Following PRISMA 2020, the screening pipeline proceeded in four stages: identification, screening, eligibility assessment, and inclusion. Each step was reviewed independently by two reviewers; conflicts were resolved by a third reviewer.

A total of 412 records were identified from the four databases. After removing 45 duplicates, 367 records underwent title and abstract screening, resulting in 230 articles for full-text review. Based on the operational inclusion/exclusion criteria, 184 studies were deemed eligible for inclusion.

Of these 184 included studies, 153 peer-reviewed publications were examined in depth within the thematic analysis (Section 5.1, Section 5.2 and Section 5.3) and are summarized in the comparative tables.

The remaining 31 sources (27 peer-reviewed papers and 4 authoritative web sources) were used exclusively for contextual background and foundational definitions, primarily in Section 3 (Industry 5.0 overview) and Section 4 (AIoT technological foundations). These background sources provided policy frameworks, emerging industrial standards, and conceptual foundations not yet widely covered in the peer-reviewed PdM literature, but were not used as primary evidence for technical AIoT or predictive maintenance claims.

**Figure 1:**
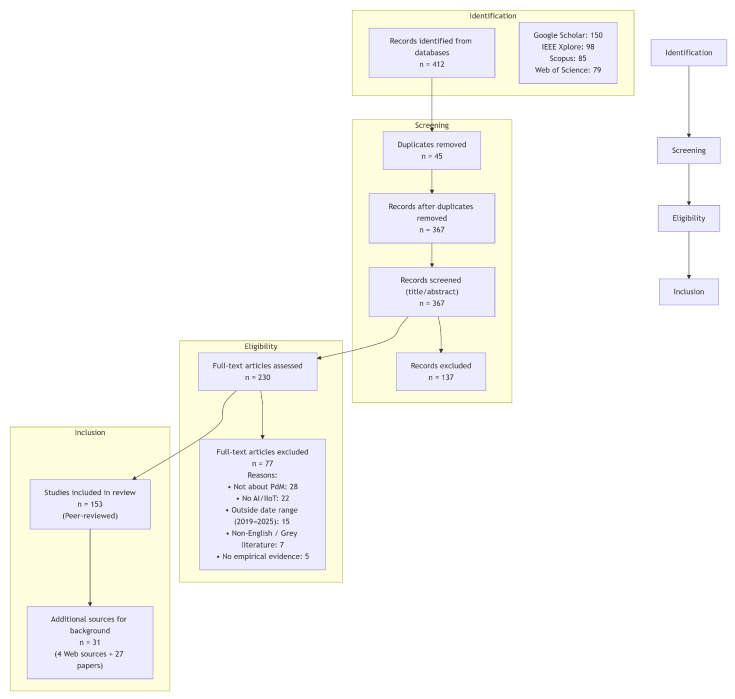
PRISMA-based literature selection process.

Figure 1 presents the complete PRISMA 2020 flow diagram with detailed counts and exclusion categories.

### 2.6. Quality Assessment

To ensure the reliability and relevance of included studies, we implemented a structured quality assessment procedure. Each full-text study was independently evaluated by two reviewers according to the following criteria:

1. Methodological Rigor: Clarity of research design, reproducibility of experiments, appropriateness of evaluation metrics, and transparency in reporting results.
2. Technical Novelty: Contribution to the state-of-the-art in AIoT, PdM, or IIoT architectures beyond incremental improvements.
3. Integration Depth: Meaningful convergence of AI and IoT components rather than superficial juxtaposition.
4. Alignment with Industry 5.0: Explicit or implicit support for human-centricity, sustainability, or resilience.
5. Real-World Applicability: Presence of case studies, industrial validation, scalability considerations, or deployment insights.

Studies were rated descriptively as High, Medium, or Low on each criterion. Discrepancies between reviewers were resolved through discussion or arbitration by a third reviewer. Studies rated Low in methodological rigor or technical novelty were excluded from the final synthesis. The assessment results informed the critical analysis in Section 5 and Section 6, ensuring that this review prioritizes robust, impactful, and practically relevant research.

### 2.7. Review Structure

**Figure 2:**
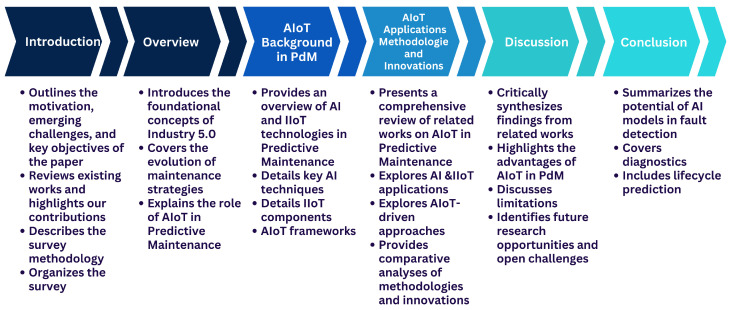
Review Roadmap.

The structure of this review follows a progressive and thematic organization aligned with this survey’s objectives (Figure 2). Following the introduction and methodology, an overview section establishes the context by discussing Industry 5.0, the evolution of maintenance strategies, and the strategic role of AIoT in predictive maintenance. The background section then elaborates on key technological enablers, including AI, the IIoT, and their integration into AIoT frameworks in PdM.

This is followed by an in-depth Related Works section, which classifies and analyzes 153 peer-reviewed articles across three major dimensions: AI techniques in predictive maintenance, IIoT-based applications across sectors such as manufacturing, transportation, and energy, and AIoT-driven approaches that bridge both fields.

Subsequently, the discussion section offers a critical synthesis of the findings, highlighting the advantages, limitations, and future research opportunities. This paper concludes by summarizing key insights and outlining directions for future work.

## 3. Overview

This section explores key concepts that shape modern industrial maintenance. It begins with an introduction to Industry 5.0, highlighting its shift toward human-centric, sustainable, and resilient manufacturing. Next, it examines the evolution of maintenance strategies, tracing the transition from reactive and preventive approaches to predictive techniques enhanced by AI. Finally, it delves into the integration of AIoT in predictive maintenance, discussing how AI-driven analytics and IoT connectivity improve fault detection, optimize maintenance schedules, and enhance industrial efficiency.

### 3.1. Industry 5.0: A Human-Centric and Resilient Paradigm

**Figure 3:**
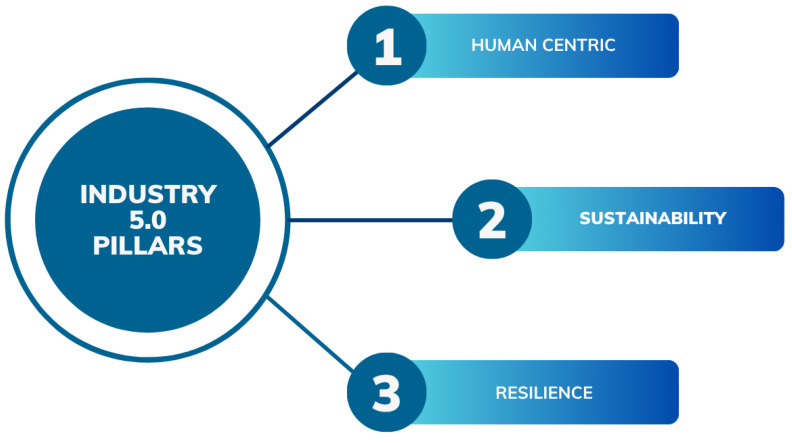
Industry 5.0 pillars adapted from the European Commission’s conceptualization [1]. Each pillar directly informs predictive maintenance objectives: (1) Human-centricity emphasizes worker safety, explainable AI, and human-in-the-loop decision support in maintenance operations; (2) Sustainability drives energy-efficient monitoring, reduced material waste through precise maintenance timing, and extended asset lifespan; (3) Resilience enables adaptive maintenance strategies, fault-tolerant architectures, and rapid recovery from disruptions through predictive analytics. AIoT-enabled PdM systems operationalize these pillars through intelligent sensing, analytics, and autonomous decision-making.

Industry 5.0 represents a shift from the technology-driven automation of Industry 4.0 to a more human-centric, resilient, and sustainable industrial model. According to the European Commission, Industry 5.0 prioritizes human well-being, sustainability, and resilience as core pillars of future industrial development [1], represented in Figure 3. This new paradigm does not replace Industry 4.0 but builds upon its digital and cyber-physical foundations, such as IoT, AI, and cloud computing, by embedding ethical values and societal goals into the production environment.

Rather than emphasizing full automation, Industry 5.0 promotes human-machine collaboration through technologies like collaborative robots (cobots), explainable AI, and immersive interfaces. These innovations empower workers to co-create value with intelligent systems, enhancing adaptability in increasingly complex production settings.

Sustainability is also a defining component, with a focus on circular economy principles, energy efficiency, and smart resource management. Resilience is achieved through distributed intelligence, decentralized control systems, and predictive capabilities that ensure system responsiveness during disruptions, such as pandemics, supply chain shocks, or extreme weather events.

In the context of industrial maintenance, Industry 5.0 strengthens the role of intelligent decision support systems, enabling more contextualized and ethical interventions. AIoT-enabled predictive maintenance, for instance, aligns closely with Industry 5.0 goals by using real-time monitoring and machine learning to minimize resource waste, reduce environmental impact, and improve worker safety. This convergence between Maintenance 4.0 and Industry 5.0 is central to the future of industrial reliability.

### 3.2. Evolution of Maintenance Strategies

As industrial systems grow in complexity, maintenance strategies have progressively evolved to improve operational reliability, minimize downtime, and extend asset lifespans. This evolution reflects a shift from reactive and schedule-based approaches to data-driven and intelligent methodologies powered by AI and IIoT.

**Figure 4:**
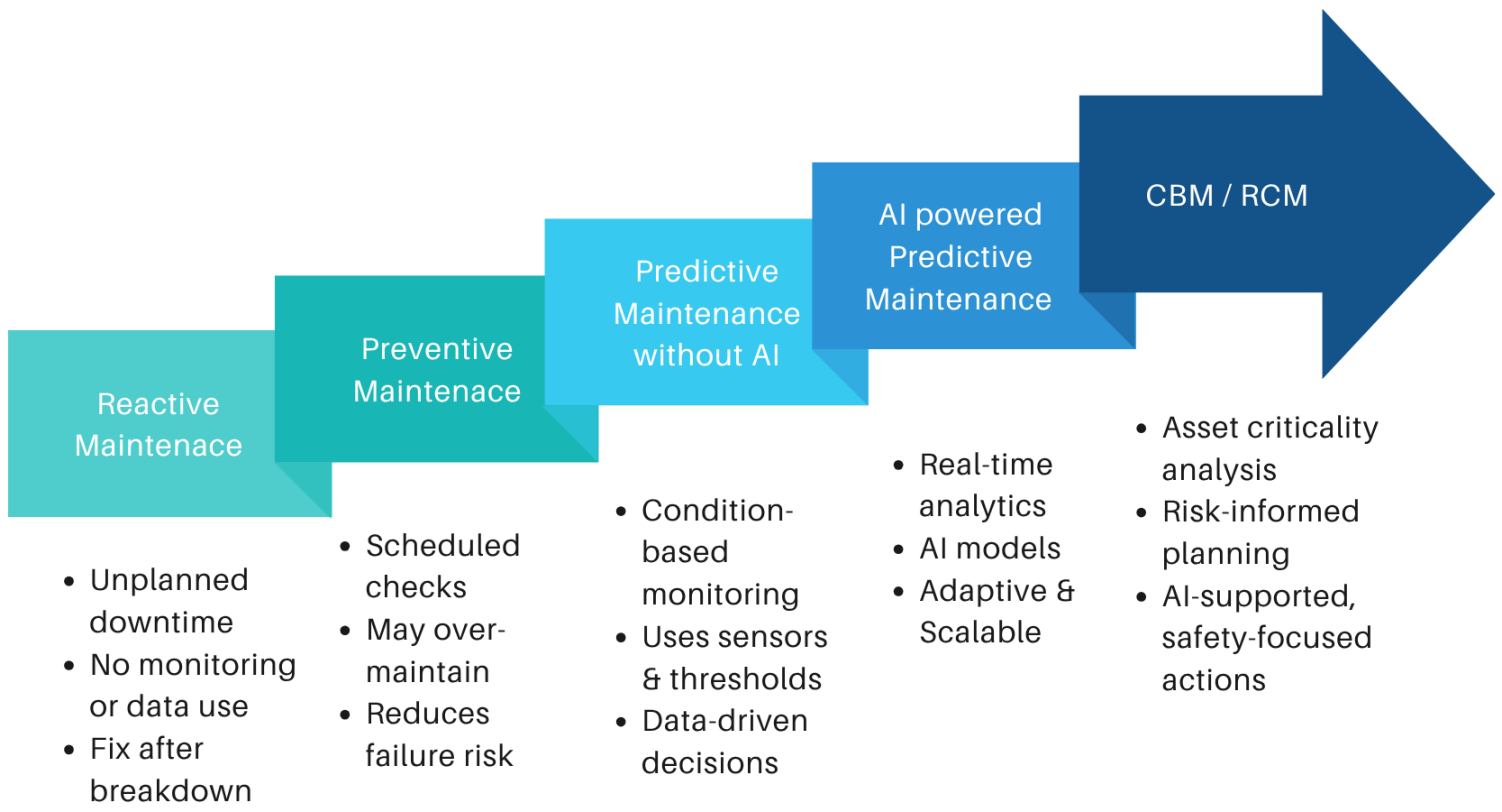
Evolution of maintenance strategies from reactive approaches to intelligent, data-driven methodologies. The progression includes: Reactive Maintenance (RM), Preventive Maintenance (PM), Predictive Maintenance without AI, AI-powered Predictive Maintenance, and advanced strategies such as Condition-Based Maintenance (CBM) and Reliability-Centered Maintenance (RCM). CBM tailors maintenance actions to actual equipment condition using real-time sensor data, while RCM prioritizes maintenance based on asset criticality, failure consequences, and safety implications.

Figure 4 illustrates the trajectory of this progression from early reactive methods to modern AI-powered predictive maintenance. Each stage introduces new capabilities in terms of monitoring precision, automation, and responsiveness.

- Reactive Maintenance (RM): This traditional approach addresses equipment only after failure occurs. While easy to implement, it often results in high repair costs, unplanned downtime, and production losses due to a lack of foresight.
- Preventive Maintenance (PM): PM schedules regular maintenance based on manufacturer recommendations or operating hours, regardless of actual equipment condition. Though it reduces surprise failures, it can lead to unnecessary interventions or overlook emerging faults.
- Predictive Maintenance without AI: This strategy uses condition-monitoring technologies, like vibration analysis, acoustic emissions, and thermography, to detect anomalies and forecast failures. While more accurate than PM, its manual interpretation limits scalability and real-time responsiveness.
- AI-powered Predictive Maintenance: Leveraging machine learning and deep learning, this approach automatically analyzes sensor data, identifies patterns, and predicts failures with high accuracy [2]. AI-powered PdM enables real-time insights, optimized maintenance scheduling, and better resource utilization, aligning with Industry 5.0 values of resilience and sustainability.
- Condition-Based and Reliability-Centered Maintenance (CBM/RCM): Emerging strategies like CBM and RCM combine real-time condition data with system-level risk analysis. CBM tailors actions to actual wear or performance decline, while RCM prioritizes maintenance based on the criticality of assets, failure consequences, and safety implications, often supported by AI-driven diagnostics.

### 3.3. AIoT in Predictive Maintenance

Artificial Intelligence of Things (AIoT) represents the convergence of AI and IoT, enabling intelligent, connected maintenance systems. In predictive maintenance, AIoT plays a pivotal role by linking real-time sensor data from physical assets with advanced analytics to anticipate failures before they occur. Unlike isolated IoT deployments or standalone AI models, AIoT facilitates continuous learning, adaptive diagnostics, and context-aware decision-making across industrial environments.

This synergy enhances conventional condition-based maintenance by embedding intelligence at various levels, from local edge devices to cloud platforms, thus supporting both timely fault detection and optimized maintenance scheduling. AI algorithms process data from IoT-enabled machinery to detect patterns, anomalies, and degradation trends that would be difficult to identify manually.

As a result, AIoT empowers maintenance strategies like predictive and reliability-centered maintenance (RCM), aligning with Industry 5.0’s emphasis on resilience, adaptability, and human-centric systems. It provides a scalable pathway to operational efficiency, reduced downtime, and data-informed decision-making across diverse industrial sectors.

## 4. AIoT Background in PdM

Artificial Intelligence and the Internet of Things have emerged as transformative forces across industries, enabling intelligent automation, real-time monitoring, and predictive capabilities. AI encompasses computational techniques, such as machine learning, deep learning, and generative models, that empower systems to extract insights from data and make autonomous decisions. Meanwhile, the Industrial Internet of Things facilitates large-scale data acquisition and connectivity through sensor networks, edge computing, and cloud platforms. This section explores the foundational principles of AI and IIoT individually, then introduces their convergence under the AIoT paradigm. The final subsection highlights how AIoT enables advanced predictive maintenance systems by bridging intelligent analytics with industrial sensing infrastructures.

### 4.1. Artificial Intelligence

#### 4.1.1. The Evolution of AI in Predictive Maintenance

Artificial intelligence has revolutionized predictive maintenance by enabling proactive failure detection and efficient resource allocation. Early approaches, such as support vector machines, decision trees, and basic neural networks, established the foundation for fault classification and equipment health monitoring [3]. However, the growing complexity and volume of industrial data in modern manufacturing demand more scalable and adaptive techniques.

Machine learning plays a central role in predictive maintenance, automating tasks like fault classification, estimation of remaining useful life, and anomaly detection. Supervised learning leverages labeled datasets for precise predictions, while unsupervised methods uncover patterns in unlabeled sensor data [4]. Reinforcement learning optimizes maintenance schedules through dynamic decision-making, showing potential in adaptive industrial applications [5].

Recent advances in deep learning, particularly convolutional neural networks and recurrent neural networks such as long short-term memory units, have improved predictive maintenance by modeling spatial and temporal sensor data. Convolutional networks excel in analyzing vibration and thermal signals, while long short-term memory networks capture time-dependent degradation patterns [6]. However, these models face limitations in handling long-range dependencies and computational scalability.

Generative techniques further expand predictive maintenance capabilities through synthetic data generation and uncertainty quantification. Variational autoencoders and generative adversarial networks create synthetic failure data, enhancing model robustness in data-scarce environments, such as energy sector applications [7].

The Transformer model, with its self-attention mechanism, has transformed sequence modeling by efficiently capturing long-range dependencies in time-series data [8]. Variants such as Informer and the time-series Transformer outperform traditional recurrent and convolutional networks in anomaly detection, remaining useful life prediction, and fault classification, offering superior scalability for industrial internet-of-things systems [9].

Large language models, initially developed for natural language processing, are being adapted for predictive maintenance. When fine-tuned on sensor data and maintenance logs, these models enable operational insights, intervention recommendations, and natural-language interfaces for collaboration between human experts and intelligent systems [10]. Emerging techniques such as diffusion models for high-fidelity failure simulation and graph neural networks for modeling equipment degradation and sensor interactions further advance predictive maintenance by capturing complex system dependencies [11,12].

**Figure 5:**
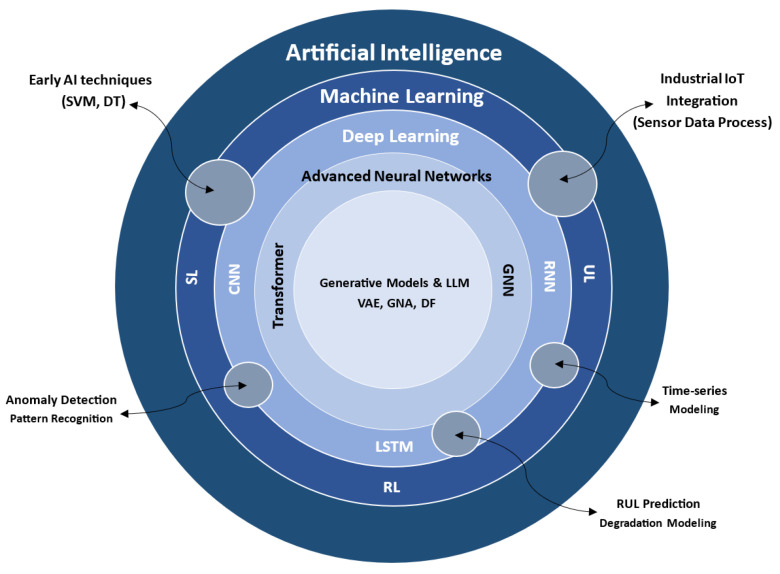
Artificial Intelligence Techniques in Predictive Maintenance.

These state-of-the-art artificial intelligence techniques align with the human-centric and adaptive objectives of modern manufacturing, fostering resilient, scalable, and intelligent predictive maintenance strategies. Figure 5 illustrates the hierarchical evolution of AI techniques in predictive maintenance, from foundational machine learning approaches to state-of-the-art generative models and large language models.

#### 4.1.2. Bridging Conventional Modeling and AI in Predictive Maintenance

While artificial intelligence has enabled data-driven approaches that excel in pattern recognition and predictive accuracy, it is important to recognize that conventional model-based techniques remain highly relevant in predictive maintenance. These physics-based or rule-based methods rely on explicit mathematical modeling of system behavior, leveraging domain expertise to simulate degradation processes, define failure thresholds, or estimate system health [13]. They are often favored for their explainability and reliability in safety-critical applications, such as those in aerospace and energy sectors, where understanding the reasoning behind predictions is critical [14].

Rather than replacing these approaches, artificial intelligence methods, particularly machine learning, can complement and enhance them. For instance, artificial intelligence can be used to learn residual patterns not captured by analytical models or to optimize parameters in physical simulations [15]. Conversely, model-based insights can guide artificial intelligence models by constraining predictions to physically plausible ranges or generating synthetic data for training when real-world faults are rare [16]. This hybrid approach, known as gray-box modeling, allows for combining the strengths of both paradigms: the interpretability and structure of physical models with the flexibility and learning capability of artificial intelligence [17].

In the context of Industrial Internet of Things systems, combining model-based and artificial intelligence-based methods enhances predictive maintenance by offering both accurate predictions and transparent reasoning. This integration supports decision-makers in industries where both trust in the system and advanced analytics are critical, particularly in sectors like manufacturing, energy, and aerospace [18].

### 4.2. Industrial Internet of Things

The Industrial Internet of Things (IIoT) refers to the integration of interconnected, sensor-equipped devices within industrial environments to enable real-time monitoring, data collection, and intelligent decision-making. In predictive maintenance, IIoT serves as the backbone infrastructure that supports continuous condition monitoring, fault detection, and failure prediction through seamless data flow from physical assets to analytics platforms [19].

**Figure 6:**
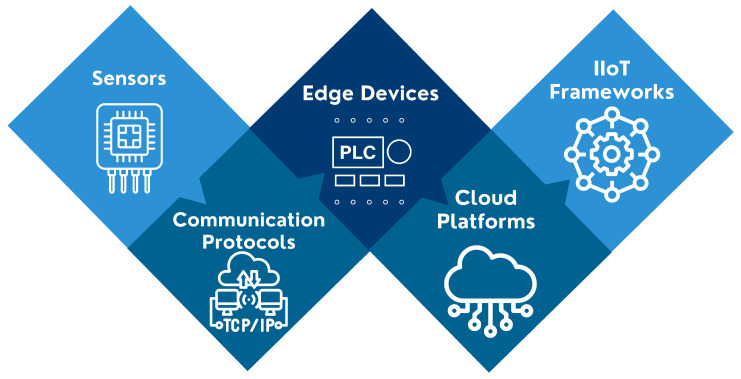
IIoT Components.

IIoT enables this by combining sensing technologies, communication protocols, edge analytics, and cloud computing to shift from reactive maintenance strategies to proactive and intelligent maintenance operations. Figure 6 provides a visual overview of the key technological components supporting IIoT-enabled PdM systems.

#### 4.2.1. Sensors

Sensors are a cornerstone of IIoT-based predictive maintenance, acting as the primary data source for assessing equipment health. They enable real-time monitoring, diagnostics, and anomaly detection across a wide range of industrial systems [20]. In PdM, sensors detect early signs of degradation, minimizing downtime and optimizing maintenance schedules [21].

Vibration sensors monitor rotating machinery to detect imbalances, misalignment, or bearing faults. Temperature sensors identify overheating, lubrication failures, and excessive friction in systems like boilers and transformers. Pressure sensors ensure that fluid and gas systems operate within safe thresholds, helping to detect leaks or pump failures. Electrical sensors, such as those measuring current or power consumption, track anomalies in motors and circuits.

Sensor fusion enhances predictive capabilities by integrating data from multiple sensor types, improving detection accuracy, reducing false alarms, and providing a more holistic view of system behavior [22,23].

#### 4.2.2. Communication Technologies, Protocols, and Standards

Efficient and reliable communication technologies form the backbone of the Industrial Internet of Things, enabling seamless data exchange between sensors, edge devices, cloud platforms, and industrial control systems. Given the diverse and often harsh industrial environments, IIoT networks require robust connectivity solutions that ensure low latency, high reliability, and scalability. The choice of communication technology depends on factors such as data transmission range, bandwidth requirements, power consumption, and environmental conditions [24].

Beyond communication technologies, IIoT communication relies on specialized protocols and standards that define how devices exchange data securely and efficiently. Protocols such as MQTT, OPC UA, Modbus, and PROFINET facilitate M2M communication, ensuring real-time data flow for industrial automation and PdM. Additionally, ISO/IEC 30141 and IEC 62443 provide frameworks for interoperability, reliability, and cybersecurity in IIoT environments [25].

IIoT communication technologies can be broadly categorized into wired and wireless solutions (Table 2). Wired networks, such as Ethernet-based industrial protocols and fieldbus systems, offer high-speed, secure, and interference-resistant data transmission, making them ideal for mission-critical industrial operations. However, wireless technologies, including Wi-Fi, Bluetooth, Zigbee, LoRaWAN, and cellular networks (4G, 5G), provide greater flexibility, allowing for remote monitoring and large-scale sensor deployments [25]. Each of these technologies has distinct advantages and trade-offs, influencing their suitability for specific IIoT applications, including predictive maintenance.

For PdM, continuous monitoring and real-time data transmission are essential to detect early signs of equipment degradation. Low-latency networks, such as 5G and industrial Wi-Fi, support high-speed data transfer for instant anomaly detection, while LPWANs, like LoRaWAN, enable long-range, energy-efficient communication for distributed asset monitoring. Similarly, industrial communication protocols like MQTT and OPC UA (Table 3) enable lightweight, scalable, and secure data exchange between devices and cloud analytics platforms, supporting advanced analytics, predictive modeling, and automated maintenance responses [26].

#### 4.2.3. Edge Analytics

Edge analytics plays a vital role in IIoT-based PdM by enabling local data processing at or near the source of data generation. Instead of transferring all sensor data to the cloud, edge analytics filters, preprocesses, and analyzes data in real time, reducing bandwidth usage and latency while enhancing system resilience in environments with limited connectivity [27].

Edge analytics encompasses a variety of devices such as industrial gateways, programmable logic controllers (PLCs), industrial PCs, and edge AI hardware. These devices enable predictive capabilities at the edge, including real-time fault detection and early warning systems Table 4. Gateways aggregate and translate protocol data, edge computers execute advanced models, PLCs implement deterministic control with condition monitoring, and single-board computers like the Raspberry Pi provide low-cost flexibility for edge AI deployment [28,29].

#### 4.2.4. Cloud Platforms

Cloud platforms offer centralized infrastructure for large-scale data storage, advanced analytics, and AI integration. In PdM, they enable multi-site data aggregation, long-term trend analysis, and deployment of predictive models across fleets of assets [30].

Platforms like AWS IoT, Microsoft Azure IoT, and Google Cloud IoT provide services such as real-time dashboards, predictive modeling, anomaly detection, and secure device management, supporting scalable and adaptive PdM strategies.

#### 4.2.5. IIoT Frameworks for Predictive Maintenance

Several IIoT frameworks facilitate the collection, transmission, and analysis of data from industrial equipment, which is crucial for monitoring the health of assets and predicting potential failures before they occur. Below, we present Table 5, which summarizes some key IIoT frameworks, their descriptions, applications in PdM, strengths, and challenges.

### 4.3. AIoT Integration for Predictive Maintenance

AIoT represents the strategic convergence of AI and IIoT technologies to create intelligent, connected, and autonomous industrial systems. In PdM, this integration enhances the capability of industrial assets to self-monitor, detect anomalies, and optimize maintenance decisions through continuous learning.

A typical AIoT architecture for PdM comprises three tightly coupled layers. At the sensing layer, smart industrial equipment is outfitted with a range of sensors (e.g., temperature, vibration, pressure) that generate real-time condition data. This data is transmitted to edge computing nodes that handle initial preprocessing tasks, such as noise filtering, feature extraction, or lightweight inferencing, enabling low-latency responses to critical conditions.

The processed data then flows into centralized platforms (often fog or cloud-based) where more computationally intensive AI models are deployed. These models leverage historical trends, equipment profiles, and contextual information to generate maintenance predictions, classify failure types, and recommend corrective actions. The system is typically supported by feedback loops that continuously retrain AI models using new operational data, allowing for adaptive behavior over time.

By enabling multi-layer intelligence, from the edge to the cloud, AIoT allows predictive maintenance strategies to scale across large industrial deployments while maintaining responsiveness. Furthermore, AIoT provides the technological backbone for implementing advanced maintenance approaches such as condition-based maintenance and reliability-centered maintenance, where both real-time equipment status and strategic risk assessments drive decisions.

**Figure 7:**
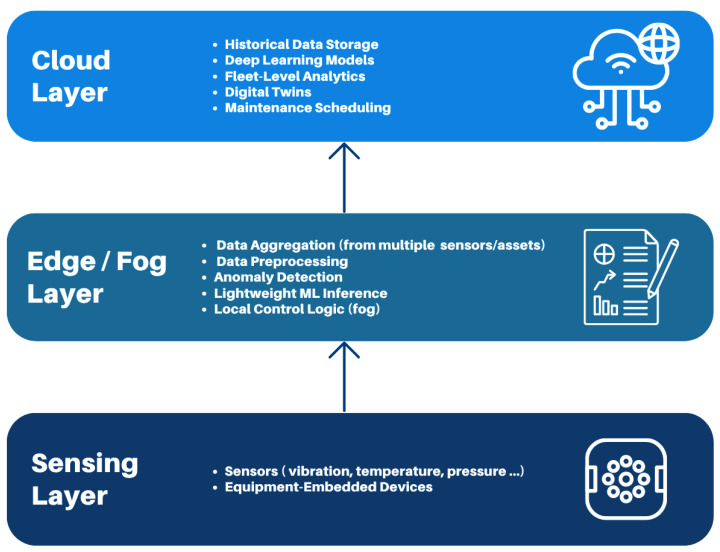
General AIoT Architecture for PdM.

A general AIoT architecture for PdM is presented in Figure 7.

## 5. AIoT-Based Predictive Maintenance: Applications, Methodologies, and Innovations—Related Works

The convergence of artificial intelligence and the Internet of Things, collectively termed AIoT, has redefined predictive maintenance by enabling intelligent, data-driven decision-making across industrial domains. This section presents a structured review of AIoT-driven predictive maintenance, encompassing three key dimensions: AI applications, including fault detection and diagnosis, remaining useful life prediction, and predictive analytics for maintenance scheduling; IIoT applications, highlighting sector-specific implementations in manufacturing, transportation, and energy; and AIoT-enabled methodologies, featuring evolving paradigms such as AI-enhanced condition-based maintenance, prognostics and health management, and prescriptive maintenance, as well as next-generation innovations like Edge–Cloud collaboration, federated learning, and digital twins. By synthesizing techniques, architectures, and real-world deployments, this review illustrates how AIoT bridges the gap between data acquisition and actionable insights, fostering more resilient, adaptive, and sustainable maintenance ecosystems.

### 5.1. AI Applications in PdM

AI has become a fundamental enabler of PdM, empowering industries to transition from traditional maintenance paradigms to intelligent, predictive strategies. By harnessing advanced techniques, including Machine Learning (ML), Deep Learning (DL), Reinforcement Learning (RL), and generative models, PdM systems can analyze high-dimensional sensor data, detect anomalies, and forecast potential failures with unprecedented accuracy.

Methodological Note on Performance Comparability: The following synthesis is based on peer-reviewed studies that met our inclusion criteria. It is important to emphasize that performance metrics (e.g., accuracy, F1-score, RMSE) reported across different studies cannot be directly compared, as they are derived from diverse datasets, operating conditions, and failure definitions. The objective of this section is to provide a structured overview of methodological trends and capabilities rather than a ranked comparison of algorithms.

**Figure 8:**
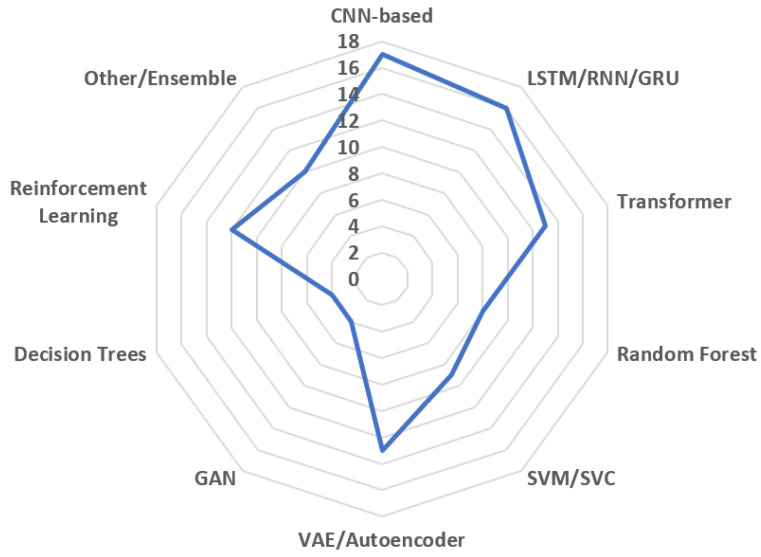
Distribution of AI model types across the studies reviewed in Section 5.1.

As illustrated in Figure 8, convolutional and recurrent neural networks dominate the current landscape of AI-driven PdM.

#### 5.1.1. Fault Detection and Diagnosis

Fault detection and diagnosis (FDD) play a fundamental role in predictive maintenance by identifying and analyzing faults in industrial systems before they lead to failures. While comprehensive reviews exist for domain-specific applications such as railway point machines [31] and for general data-driven diagnosis methodologies [32], this subsection focuses specifically on AI-driven FDD methods enabled by the AI paradigm. These methods enhance reliability, reduce downtime, and optimize maintenance strategies by leveraging advanced techniques such as machine learning, deep learning, and anomaly detection. Traditional model-based approaches, including analytical redundancy and Kalman filters, are now complemented by AI-powered data-driven methods that analyze sensor data for real-time fault prediction. Additionally, signal-based techniques, such as vibration and acoustic analysis, along with knowledge-based systems like fuzzy logic and expert systems, contribute to comprehensive fault detection. With the integration of AIoT, FDD solutions now utilize real-time monitoring, cloud analytics, and edge computing to improve decision-making and ensure proactive maintenance. The following works explore various AI methodologies applied to FDD in PdM.

Building on the importance of FDD in predictive maintenance, supervised learning has gained significant attention due to its effectiveness in classifying faults and predicting failures based on labeled datasets to learn patterns associated with normal and faulty operating conditions. The following studies examine the performance of different SL models in PdM.

Rai and Wollega evaluated seven machine learning models, including Logistic Regression, LDA, QDA, SVC, Random Forest, Gradient Boosting, and ANN, and found that SVC outperformed others in precision-recall metrics, making it highly effective for failure prediction, while LDA and QDA showed lower accuracy [33]. Similarly, Tarik and Jebari reviewed Decision Trees, Random Forests, SVM, k-NN, and ANN, emphasizing the high accuracy of Random Forests and ANN in modeling complex relationships, though SVM was noted for its computational demands [34]. Ouadah et al. focused on Random Forests, Decision Trees, and k-NN, revealing that Random Forests excelled on smaller datasets, while k-NN was more effective with larger datasets, underscoring the importance of dataset characteristics in algorithm selection [35]. Levin further advanced this discourse by evaluating Random Forest, GBM, and DNNs, with DNNs demonstrating superior performance in industrial settings, particularly in identifying key failure predictors like vibration levels and operational hours [36]. In a related study, Muhammed et al. collected 3478 data points, including gas levels, temperature, and humidity, and evaluated the performance of four ML algorithms, Decision Tree, Gaussian Naive Bayes, Gaussian Process Classifier, and Support Vector Machine, using 5-fold cross-validation for hyperparameter optimization. Among these, the Gaussian Process Classifier achieved the highest performance, with an accuracy of 99.56%, a precision of 0.978, a recall of 0.989, an F1 score of 0.983, and an AUC of 0.99 [37]. Collectively, these studies underscore the potential of SL in enhancing PdM strategies, while also highlighting challenges such as computational demands, dataset suitability, and the need for specialized expertise.

Table 6 presents a structured comparison of studies on supervised learning approaches applied to FDD, summarizing their employed techniques, key contributions, performance improvements, and associated limitations.

In addition to supervised learning, various methodologies have been explored to address the challenges of working with unlabeled data, each contributing unique insights into fault detection and diagnostic improvements. Sergio et al. proposed a machine learning-based anomaly detection system for induction motors. Their study compared three algorithms: One-Class Support Vector Machine, Isolation Forest, and Local Outlier Factor. Among the tested methods, LOF demonstrated the best trade-off, achieving a sensitivity of 77.6% and specificity of 72.1%, with an inference time of 0.81 milliseconds [38]. Building on the potential of unsupervised learning, Amruthnath and Gupta investigated clustering and dimensionality reduction techniques for early fault detection in predictive maintenance. Their study assessed Principal Component Analysis T^2^ statistic, hierarchical clustering, K-means clustering, fuzzy C-means, and model-based clustering on vibration data from an exhaust fan. The results indicated that PCA T^2^ statistic and model-based clustering were most effective in identifying faults [39]. A distinct approach was presented by Giannoulidis et al., who developed a cold-forming press using a profile-based anomaly detection algorithm. This method constructs an operational profile from initial machine data and evaluates new data points by measuring deviations from this profile. The study emphasized that factors such as material changes and maintenance activities significantly influenced anomaly scores, reflecting the press’s dynamic behavior [40]. To further improve clustering-based fault detection, Langone et al. explored Kernel Spectral Clustering. By integrating kernel methods into spectral clustering, KSC effectively handled high-dimensional sensor data, outperforming traditional clustering techniques in accuracy and robustness [41]. Focusing on data preprocessing and quality, Nyqvist et al. propose an approach for addressing data quality issues. By incorporating dimensionality reduction techniques within the CRISP-DM framework, their method facilitated better anomaly detection and diagnostic analysis, ultimately improving maintenance decision-making [42]. In contrast, Liu et al. introduced a novel approach combining data augmentation with soft contrastive learning to enhance fault detection capabilities. Traditional Gaussian-based assumptions often limit anomaly detection models, but their proposed USD method increased sensitivity to subtle anomalies, surpassing existing techniques on industrial benchmark datasets such as SWaT, WADI, PSM, and MSL. With an average performance score of 57.5, and an enhanced version (USD*) reaching 64.4 [43]. Lastly, Mateo et al. developed an expert system designed to improve predictive maintenance for packaging machines in logistics warehouses. Their two-stage framework first employed a binary classifier to assess sensor alarms, followed by an anomaly detection layer to refine its probabilistic outputs. Three anomaly detection methods, OC-SVM, Minimum Covariance Determinant, and a Majority Voting Ensemble, were evaluated, with the ensemble approach yielding the highest accuracy [44]. Together, these studies illustrate the growing role of unsupervised learning in predictive maintenance, highlighting various techniques, from clustering-based methods and anomaly profiling to contrastive learning and hybrid expert systems. Each approach contributes to the broader goal of early fault detection, improving reliability and reducing maintenance costs in next-generation industrial systems.

Table 7 presents a structured comparison of studies on unsupervised learning approaches applied to FDD, summarizing their employed techniques, key contributions, performance improvements, and associated limitations.

Expanding on the advancements in unsupervised learning, reinforcement learning has emerged as a powerful tool in fault detection due to its ability to adaptively optimize system performance and enhance fault diagnosis accuracy under dynamic and uncertain conditions. Zaccaria et al. introduce a novel Active Fault Detection approach, decoupling Passive Fault Detection from control input design. Their method, FIERL, uses Constrained Reinforcement Learning to optimize control strategies, improving fault detection efficiency while meeting system constraints. FIERL excels in complex fault scenarios, including continuous fault mode variations, and demonstrates robustness, adaptability to unseen fault dynamics, and faster, more accurate fault diagnosis compared to traditional methods, advancing AI-driven predictive maintenance [45]. Gensheng Qian and Jingquan Liu tackle fault diagnosis in rotating machinery within nuclear power plants, where rare fault occurrences due to strict safety standards result in limited data. They propose two deep reinforcement learning models that combine deep learning’s feature extraction with RL’s interactive learning, achieving over 99% diagnosis accuracy with small datasets. These models outperform traditional methods like SVM, CNN, and GRU, highlighting RL’s potential to enhance NPP safety and reliability in data-scarce environments [46]. Ding et al. present a Deep Q-Network-based framework for fault diagnosis in rotating machinery, modeling the problem as a Markov Decision Process. The DQN agent interacts with the system, using vibration signals as state representations and receiving rewards based on classification accuracy. This approach achieves high accuracy in identifying fault types and health states, outperforming traditional methods and proving robust under variable operating conditions, showcasing RL’s potential for adaptive predictive maintenance [47]. Wang et al. address actuator faults in industrial systems using an RL-based approach. They propose a state-space model incorporating tracking dynamics and state increment information, deriving value and Q-functions to optimize control laws. This method expands the system’s tolerable fault range, ensuring effective control before fault elimination and improving system performance through learning. A case study on a three-capacity water tank demonstrates its superiority over traditional fault-tolerant control methods, enhancing resilience in industrial processes [48]. Collectively, these studies highlight RL’s transformative potential in fault detection across industrial applications, from actuator faults to rotating machinery in NPPs. By leveraging RL’s adaptive and interactive capabilities, these approaches improve fault detection accuracy, efficiency, and system resilience, paving the way for robust predictive maintenance strategies.

Table 8 presents a structured comparison of studies on reinforcement learning approaches applied to FDD, summarizing their employed techniques, key contributions, performance improvements, and associated limitations.

Recent advancements in predictive maintenance and fault diagnosis have leveraged deep learning techniques to enhance industrial machinery monitoring. Ferraro et al. introduced a novel methodology for anomaly detection in industrial machinery by analyzing sound data using LSTM and CNN-based autoencoders, which process continuous audio streams via a customized sliding window technique. This approach achieved low inference times and minimal memory requirements, making it suitable for real-time applications [49]. Similarly, Chang et al. proposed the Causal Disentanglement Hidden Markov Model for bearing systems, which disentangles vibration signals into fault-relevant and fault-irrelevant factors, achieving 100% accuracy in workload transfer scenarios and outperforming baseline methods like DDC and DANN [50]. Eang and Lee developed a hybrid CNN-RNN framework for fault detection in DC motor drives, demonstrating higher accuracy and faster processing times compared to existing methods [51]. Diego et al. employed autoencoder neural networks and CNNs for fault diagnosis in AC induction motors, achieving high performance in anomaly detection and classification [52]. Al-Said et al. combined CNNs and LSTMs to capture spatial and temporal patterns in sensor data, achieving F-Scores of 92% and 97% for binary and multiple classification tasks, respectively [53]. Yan et al. addressed multi-fault diagnosis in rotating machinery using a hybrid DCNN-SVM approach, improving diagnostic accuracy to 98.71% by integrating expert knowledge [54]. Choudhary et al. utilized thermal imaging and CNNs for non-invasive bearing fault diagnosis, demonstrating CNNs’ superiority over ANNs in feature extraction [55]. Chen et al. proposed a 1D-CNN optimized via reinforcement-learning-based NAS for fault detection, achieving superior accuracy and interpretability [56]. Lastly, Kumar et al. introduced an advanced CNN-based model for bearing defect diagnosis, achieving over 99.9% accuracy with optimized computational efficiency [57]. These studies collectively highlight the effectiveness of deep learning in improving predictive maintenance and fault diagnosis across diverse industrial applications.

Table 9 presents a structured comparison of studies on deep learning approaches applied to FDD, summarizing their employed techniques, key contributions, performance improvements, and associated limitations.

Generative AI leverages advanced models such as transformers, GANs, and VAEs to enhance fault detection in predictive maintenance. Transformers analyze time-series sensor data to identify anomalies and predict potential failures, GANs generate realistic fault scenarios to improve model robustness, and VAEs learn latent representations of normal machine behavior to detect subtle deviations. Several studies have demonstrated the effectiveness of these techniques. Van et al. address the challenge of limited fault samples in machine fault detection by proposing the use of GANs to generate synthetic fault signals, significantly improving model performance with accuracies of 99.41% when combining 20% real data with synthetic data and 93.1% using only synthetic data [58]. Wenqian Jiang et al. tackle imbalanced time series data in industrial fault diagnosis with a GAN-based framework, achieving 100% accuracy on the CWRU and a laboratory dataset by using an encoder-decoder-encoder architecture and anomaly scoring based on latent losses [59]. Rani et al. introduce the Generative Adversarial Wavelet Neural Operator, combining Wavelet Neural Operators and GANs for fault detection in multivariate time series, demonstrating high accuracy [60]. Li et al. propose Graph Wavelet Autoencoder and Graph Wavelet Variational Autoencoder models, leveraging spectral graph wavelet transforms for multiscale feature extraction, achieving a 3–4% performance improvement on fuel control and acoustic valve datasets [61]. Yaqiong Lv et al. enhance VAEs for rolling bearing fault detection, achieving 98.5% accuracy on the CWRU dataset by combining MSE and KL divergence in the loss function and incorporating dynamic threshold optimization [62]. VAEs are further optimized in fault diagnosis through an optimized stacked variational denoising autoencoder, improving noise resilience and achieving 97% accuracy in fault clustering [63]. Vijai et al. introduce a BiLSTM-VAE model with a dynamic loss function, achieving 98% accuracy on the SKAB and TEP datasets, outperforming traditional methods in handling imbalanced data [64]. Jakubowski et al. investigate VAEs for unsupervised anomaly detection, achieving F1 scores of 0.68 and 0.72 on the Hot Rolling Mill and C-MAPSS datasets, highlighting the need for further improvements for industrial applications [65]. Ahmed et al. propose a VAE-LSTM hybrid model for fault detection, achieving a 95% fault detection rate on the Tennessee Eastman Process dataset, outperforming PCA and standalone LSTM [66]. Guo et al. present a PSO-ConvLSTM-Transformer model for wind turbine blade icing fault detection, achieving 98.75% accuracy through feature engineering and hyperparameter optimization [62]. Wong et al. and Jin et al. explore transformer-based models for fault diagnosis in rotating machinery, achieving 98.5% and 99.5% accuracy on benchmark datasets, respectively, demonstrating their robustness and interpretability [67,68]. Wang et al. propose a Transformer Neural Network for power transformer fault diagnosis, achieving 96.2% accuracy and rapid fault response times [69]. Wu et al. and Saeed et al. highlight the effectiveness of transformer-based models in fault detection and classification, achieving 98% accuracy and outperforming traditional methods like CNNs and LSTMs [70,71]. Kang et al. introduce a Transformer model with inter-variable attention for anomaly detection in multivariate time series, achieving state-of-the-art performance on benchmark datasets [72]. Xanthi et al. combine LSTM-Autoencoders and Transformer Encoders for predictive maintenance, achieving an F1-score of 0.92 in anomaly detection and high precision in failure prediction [73]. These studies collectively demonstrate the effectiveness of advanced deep learning techniques, including GANs, VAEs, and transformers, in improving fault detection and diagnosis across various industrial applications.

Table 10 presents a structured comparison of studies on generative AI approaches applied to Fault Detection and Diagnosis, summarizing their employed techniques, key contributions, performance improvements, and associated limitations.

#### 5.1.2. Remaining Useful Life Prediction

Remaining Useful Life prediction is a cornerstone of predictive maintenance, enabling precise estimation of an asset’s operational time before failure. AI-driven RUL models enhance prognostics by integrating machine learning, deep learning, and time-series forecasting to capture complex degradation dynamics. Traditional physics-based approaches, such as fatigue modeling and stochastic degradation processes, are increasingly augmented by AI-driven data-centric methods that extract latent failure patterns from sensor streams. Hybrid methodologies, fusing physics-informed and data-driven techniques, alongside probabilistic uncertainty quantification, further refine RUL estimation. With AIoT integration, real-time data fusion, edge analytics, and cloud-based prognostics drive more adaptive and proactive maintenance strategies. The following works examine cutting-edge AI techniques applied to RUL prediction in PdM.

Building on the foundation of AI-driven RUL prediction, machine learning has emerged as a critical tool for assessing system degradation and anticipating failures in predictive maintenance. Unlike traditional threshold-based maintenance, ML models utilize historical and real-time data to identify patterns, optimize maintenance schedules, and minimize unplanned downtime. Various ML approaches, including supervised, unsupervised, and hybrid methods, have been employed to enhance RUL prediction accuracy.

Chelmiah et al. utilize vibration signal analysis with Short-Time Fourier Transform and Envelope Analysis for feature extraction, followed by linear and non-linear banding for compression, employing SVM and k-Nearest Neighbour for wear-state classification. Their best RUL prediction model achieved 74.3% accuracy with a normalized Mean Absolute Error of 0.08 [74]. Sharma et al. develop a framework applying six ML models to aviation engine datasets, where LightGBM outperformed others with the lowest RMSE values across multiple datasets, achieving an AUC of 89% [75]. Chen et al. propose a hybrid model combining Support Vector Regression and Long Short-Term Memory, optimized using a modified grey wolf algorithm and a risk-averse cost function, effectively reducing RUL overestimation and improving real-time applicability [76]. Bernar et al. assess multiple models, including RF, XGBoost, Multilayer Perceptron, and SVR, demonstrating RF’s superior performance in preventing 42% of production line failures [77]. Li et al. integrate regression models with artificial neural networks trained on vibration signal features, successfully capturing degradation trends in wind turbine and test rig case studies [78]. Behera et al. emphasize ensemble learning, showing Gradient Boosted Trees achieving 93.91% accuracy and RF 91.78%, with RF offering computational efficiency [79]. Maulana et al. enhance explainability in aircraft engine prognostics through Bayesian filtering, achieving a 34.5–55.6% RMSE improvement [80]. Vermelin et al. introduce a self-supervised framework leveraging unlabeled data for pre-training, reducing RMSE by 10–15% and lowering MAE while improving generalization across conditions [81]. Zhao et al. refine Unscented Kalman Filtering by integrating a Kalman Filter with a linear adaptive strategy to dynamically adjust noise terms, minimizing estimation errors and fluctuations in RUL predictions [82]. These studies collectively highlight the role of machine learning in improving RUL prediction accuracy, optimizing computational efficiency, and supporting predictive maintenance strategies. However, limitations persist, including data dependency, overfitting risks, and interpretability challenges.

Table 11 presents a structured comparison of studies on machine learning approaches applied to Remaining Useful Life (RUL) Prediction, summarizing their employed techniques, key contributions, performance improvements, and associated limitations.

Expanding on the capabilities of machine learning, deep learning models excel in Remaining Useful Life prediction by automatically extracting complex degradation patterns from high-dimensional sensor data, overcoming the limitations of traditional statistical and machine learning methods that require extensive manual feature engineering. This section reviews recent advancements in deep learning approaches for RUL prediction, highlighting their methodologies, contributions, and performance improvements.

Hosseinli et al. tackled the challenge of RUL estimation under variable speed conditions using a Context-aware Domain Adversarial Neural Network. By encoding speed and time as one-dimensional vectors and leveraging a phenomenological model to generate synthetic data simulating resonance frequency crossings, CA-DANN mitigated domain shifts, improving RUL prediction accuracy and robustness to speed variations [83]. Similarly, Fu et al. proposed a CNN-LSTM model for RUL estimation of low-voltage AC contactors, where feature extraction from three-phase voltage and current signals improved accuracy. The model achieved an RMSE of 54.7 and MAE of 51.8, outperforming SVM (RMSE = 88.7, MAE = 65.4), CNN (RMSE = 64.1, MAE = 56.4), and LSTM (RMSE = 58.1, MAE = 53.0), with a 14.7% RMSE and 8.1% MAE reduction compared to CNN alone [84].

Cheng et al. employed a CNN-based framework combined with Hilbert-Huang Transform to extract nonlinear degradation indicators for bearings, utilizing ε-support vector regression (ε-SVR) for final RUL predictions, achieving high accuracy in degradation trend estimation [85].

To enhance feature extraction efficiency, Zou et al. introduced the Separable Convolution Backbone Network (SCBNet), integrating an Adjacent Backbone Assembly Strategy to improve RUL estimation while maintaining low computational complexity [86]. Ozkat et al. focused on Unmanned Aerial Systems (UAS) propulsion failures, introducing mean peak frequency as a degradation indicator. Their LSTM-based model predicted the next five mean peak frequency values based on the last seven computed values, achieving RUL estimations of (1) 4 s with RMSE = 3.7142 Hz, (2) 10 s with RMSE = 1.4831 Hz, and (3) 10 s with RMSE = 1.3455 Hz, demonstrating its reliability in failure anticipation [87].

Chen et al. developed the Multi-Scale Wide-kernel Residual Long-Term Recurrent Convolutional Network (MSWR-LRCN), integrating multi-scale feature fusion, wide first-layer kernels, and an attention-based residual shrinkage unit, leading to superior noise reduction and improved prediction accuracy [88].

For complex multi-sensor environments, Ma et al. applied a Stacked Sparse Autoencoder (SSAE) with logistic regression to extract and fuse degradation features, optimizing performance through grid search [89]. Xia et al. introduced a two-stage deep neural network (DNN) leveraging a denoising autoencoder for health stage classification followed by shallow neural networks for RUL estimation [90].

Kang et al. combined MLP with PCA for dimensionality reduction and interpolation for missing data, achieving HI Training MSE values from $3.36\times 10^{-2}$ to $4.25\times 10^{-2}$, Training RUL MSE between 21 and 94, and Validation RUL MSE ranging from 509 to 1427 across datasets [91]. Rath et al. integrated a BiLSTM network with a novel two-stage feature selection algorithm and change point detection, demonstrating a 27.8% improvement in prediction accuracy on the C-MAPSS dataset [92].

Further advancements include Qin et al.’s Temporal Deep Degradation Network (TDDN), which applies a 1D CNN with an attention mechanism to extract temporal degradation patterns for complex machinery [93]. Wang et al. incorporated Bayesian-optimized MLP with Random Forest feature selection and single exponential smoothing (SES), achieving a 6.1% RMSE reduction on the selected dataset [94].

Despite these advances, limitations persist. Many approaches rely on extensive labeled datasets, which are costly to obtain. While domain adaptation techniques like CA-DANN address domain shifts, real-world conditions often introduce additional variability. Models leveraging hybrid architectures (e.g., CNN-LSTM, SSAE) enhance feature extraction but increase computational complexity. Additionally, generalization to unseen operating conditions remains a challenge, necessitating further exploration into adaptive learning and self-supervised methods. Future research should focus on reducing dependency on large labeled datasets, improving model interpretability, and enhancing robustness to varying operational conditions.

Table 12 presents a structured comparison of studies on deep learning approaches applied to Remaining Useful Life (RUL) Prediction, summarizing their employed techniques, key contributions, performance improvements, and associated limitations.

In addition to traditional deep learning approaches, generative AI methods, such as Variational Autoencoders (VAEs) and Transformer-based architectures, enable unsupervised learning of latent representations, improving predictive accuracy and adaptability to noisy and high-dimensional sensor data. These models facilitate reliable prognostics by capturing temporal dependencies, structural relationships, and degradation trends, thus playing a crucial role in industrial applications.

Costa et al. propose a Recurrent Variational Autoencoder (RVAE) that combines RNN with VAE, effectively capturing temporal dependencies and uncertainties in high-dimensional sensor data, outperforming traditional machine learning and deep learning models [95]. Similarly, Zhao et al. utilize VAE for feature extraction, followed by a regression model for RUL estimation, demonstrating superior performance in handling noisy industrial data [96]. Xu et al. introduce CMG-VAE, integrating Temporal Convolutional Networks (TCN) and Graph Representation Learning to model structural relationships in spacecraft telemetry data, achieving a 24% reduction in RMSE over baselines [97]. Huang et al. enhance VAE with Generative Adversarial Networks (GANs) and LSTM, leveraging a Gaussian Mixture Model and feature selection techniques to improve degradation trend learning without predefined failure thresholds [98]. Yi Qin et al. develop DTC-VAE, incorporating degradation-trend constraints and a Macroscopic-Microscopic-Attention LSTM (MMA-LSTM), leading to more reliable RUL predictions in rotary machinery [99].

Beyond VAE-based models, Ogunfowora explores Transformer architectures, introducing an Encoder-Transformer model inspired by Large Language Models (LLMs), achieving a 137.65% performance improvement over previous approaches [100]. Li et al. propose DSFormer, a Transformer-based model incorporating a Dual-Attention Module, TCN, and a Feature Decomposition Module, demonstrating a 3.2% improvement in RMSE and 2.5% in Score metrics [101]. Likewise, Zhang et al. present DAST (Dual Aspect Self-Attention Transformer), which leverages parallel encoders for cross-sensor and temporal feature extraction, outperforming state-of-the-art models in multi-sensor time-series data processing [102]. Fan et al. introduce STAR, integrating a two-stage attention mechanism to address temporal and sensor-wise dependencies, achieving RMSE values of 10.61 (dataset1), 13.47 (dataset2), 10.71 (dataset3), and 15.87 (dataset4), proving effective across diverse fault modes and operating conditions [103]. Chen et al. develop SAConvFormer, which combines a spatial attention-enhanced CNN with a Transformer network to analyze raw vibration data, achieving superior RUL prediction accuracy based on RMSE and MAE metrics [104]. Lastly, Zhang et al. propose LECformer, incorporating Local Enhanced Channel Self-Attention (LECSA) to dynamically weight sensor channels for improved long-term dependencies and spatial correlation modeling [105].

While these generative models have demonstrated remarkable improvements in accuracy, robustness, and uncertainty quantification, challenges remain in computational complexity, interpretability, and real-time applicability, necessitating further research to optimize deployment in industrial environments.

Table 13 presents a structured comparison of studies on generative AI approaches applied to Remaining Useful Life (RUL) Prediction, summarizing their employed techniques, key contributions, performance improvements, and associated limitations.

#### 5.1.3. Predictive Analytics and Maintenance Scheduling

Predictive analytics and maintenance scheduling play a pivotal role in optimizing industrial operations by dynamically adapting maintenance actions based on data-driven insights. Reinforcement Learning (RL)-based approaches have emerged as powerful tools for optimizing maintenance policies by learning from system interactions and balancing cost, reliability, and operational constraints. Unlike traditional rule-based or heuristic scheduling methods, RL frameworks leverage Markov Decision Processes (MDPs) and deep learning to autonomously refine decision-making strategies over time. The integration of model-free and model-based RL techniques enables adaptive scheduling tailored to evolving system conditions. The following works explore state-of-the-art RL methodologies applied to predictive analytics and maintenance scheduling in PdM.

Maomao et al. integrate RL into multistage production systems, combining a state-based model with approximate dynamic programming to optimize maintenance decisions, reducing system costs by 9.68% compared to the state-based policy (SBP), 39.07% compared to the time-based policy (TBP), and 39.56% compared to the greedy policy (GP), while improving system throughput by over 9% [106]. Similarly, Rodríguez et al. develop a multi-agent RL framework where agents with partial observations dynamically coordinate maintenance scheduling, reducing machine downtime by approximately 75%, demonstrating its effectiveness over corrective and preventive maintenance [107]. Andriotis and Papakonstantinou address the challenges of uncertainty, historical dependencies, and long-term resource constraints by integrating constrained Partially Observable Markov Decision Processes (POMDPs) with multi-agent DRL. Their framework, incorporating Bayesian inference, decentralized control, state augmentation, and Lagrangian relaxation, outperforms traditional baselines in risk-aware maintenance strategies [108]. Zhou et al. propose Hierarchical Coordinated Reinforcement Learning (HCRL) for large-scale multicomponent systems, overcoming the “curse of dimensionality” faced by MDPs through a hierarchical agent coordination structure. Their method is validated through a natural gas plant system and a 12-component series system under dependent competing risks, where it outperforms deep RL methods [109]. Nooshin et al. introduce a Deep Q-Network (DQN)-based maintenance model, formulating the problem as an MDP with degradation modeled via a gamma process. Unlike traditional discretization-based methods, their DRL approach directly learns optimal policies, reducing maintenance costs while improving system reliability [110]. Mohammadi et al. apply DRL to rail infrastructure maintenance, optimizing maintenance and renewal planning by balancing costs, safety, and operational constraints. Their model, trained on historical data and simulations, achieves significant long-term cost savings and adaptability to uncertainties [111]. Abbas et al. develop a two-level PdM framework for turbofan engines, where an input–output Hidden Markov Model (HMM) detects failure root causes and degradation at a high level, while deep RL determines optimal maintenance policies at a lower level. This hybrid approach improves interpretability and outperforms standalone deep RL and HMM-based methods [112]. Finally, Khorasgani et al. propose an offline DRL framework for optimizing maintenance decisions without real-time environment interaction, leveraging Batch-Constrained Q-learning (BCQ) and Conservative Q-learning (CQL) to mitigate distributional shifts and handle noisy data. Their approach outperforms rule-based methods in cost reduction and reliability, making it a practical choice for industries transitioning to predictive maintenance without requiring costly real-time experimentation [113].

Despite the significant advancements in RL-driven predictive maintenance, several challenges remain. Many RL-based models require large amounts of high-quality training data, which may not always be available in industrial settings. Additionally, real-world industrial environments often involve complex interdependencies and evolving operational constraints that are difficult to capture in training simulations.

Table 14 presents a structured comparison of studies on reinforcement learning (RL) approaches applied to Predictive Analytics and Maintenance Scheduling, summarizing their employed techniques, key contributions, performance improvements, and associated limitations.

#### 5.1.4. Summary and Comparative Analysis of AI Techniques for PdM

The comprehensive review of AI applications for predictive maintenance reveals distinct patterns of effectiveness, applicability, and trade-offs across different maintenance objectives.

Fault Detection and Diagnosis (FDD) demonstrates the broadest range of AI applicability, with each approach offering distinct advantages under specific conditions. Supervised learning methods, particularly ensemble techniques like Random Forests and advanced models such as DNNs and Gaussian Process Classifiers, achieve the highest accuracy (>99%) when ample labeled fault data is available [36,37]. However, in real industrial settings where labeled fault samples are scarce, unsupervised and self-supervised approaches provide more practical deployment paths. Profile-based anomaly detection and contrastive learning methods show particular promise for adapting to dynamic operational conditions without extensive labeling [40,43]. Reinforcement learning approaches, while computationally intensive, offer unique advantages for adaptive fault detection in systems with complex control interactions [45,46].

Remaining Useful Life (RUL) Prediction exhibits a clear evolution from traditional machine learning to deep and generative approaches. While ML methods like LightGBM and ensemble techniques provide solid baselines with good interpretability [75,79], deep learning models—particularly hybrid architectures combining CNNs with LSTMs or attention mechanisms—consistently achieve superior accuracy by capturing complex temporal degradation patterns [84,92]. The emergence of generative approaches like VAEs and Transformers addresses critical challenges in uncertainty quantification and domain adaptation [95,101], though at the cost of increased computational complexity and data requirements.

Note on Emerging Techniques: It is important to contextualize the maturity of certain advanced AI techniques within the PdM landscape. While Large Language Models (LLMs) and diffusion models appear in Figure 5 as part of the generative AI evolution, their practical application in industrial PdM remains emerging and experimental. Within the scope of this review, few industrial deployments of LLMs for core maintenance tasks (e.g., interpreting maintenance logs, generating recommendations) or diffusion models for high-fidelity fault simulation were identified. These techniques are included as high-potential trends rather than established practices, reflecting their promising research directions rather than widespread industrial adoption.

Predictive Analytics and Maintenance Scheduling is uniquely served by reinforcement learning frameworks, which outperform traditional optimization methods in dynamic, multi-component environments [109,112]. The ability of RL to balance multiple objectives (cost, reliability, safety) while adapting to evolving system states makes it particularly suitable for complex industrial scheduling problems, though challenges remain in training data requirements and real-world validation.

Key Trade-offs and Practical Considerations:

- Accuracy vs. Interpretability: While deep and generative models achieve the highest accuracy, simpler ML models often provide better interpretability—a crucial factor in safety-critical applications.
- Data Requirements: The performance gap between data-hungry deep learning approaches and more data-efficient methods narrows in real-world settings where labeled fault data is limited.
- Computational Complexity: Model selection must balance predictive performance against deployment constraints, particularly for edge implementations where latency, power consumption, and model size are critical factors.
- Generalization vs. Specialization: Methods that excel on benchmark datasets may underperform when faced with domain shifts, noise, or unseen failure modes common in industrial environments.
- Maturity vs. Innovation: While foundational ML/DL methods have proven industrial track records, newer paradigms (e.g., LLMs, diffusion models, federated learning) offer innovative capabilities but require further validation in production environments.

This analysis reveals that no single AI approach dominates all PdM applications. Rather, the optimal choice depends on a careful consideration of the specific maintenance objective, data availability, computational constraints, and deployment environment—a theme that extends to the IIoT implementations and AIoT paradigms discussed in subsequent sections.

### 5.2. IIoT Applications in PdM

The Industrial Internet of Things plays a crucial role in enabling predictive maintenance by providing the necessary infrastructure for real-time data collection, processing, and communication. Through interconnected sensors, edge devices, and cloud-based platforms, IIoT allows industries to monitor equipment health, detect anomalies, and optimize maintenance strategies.

**Figure 9:**
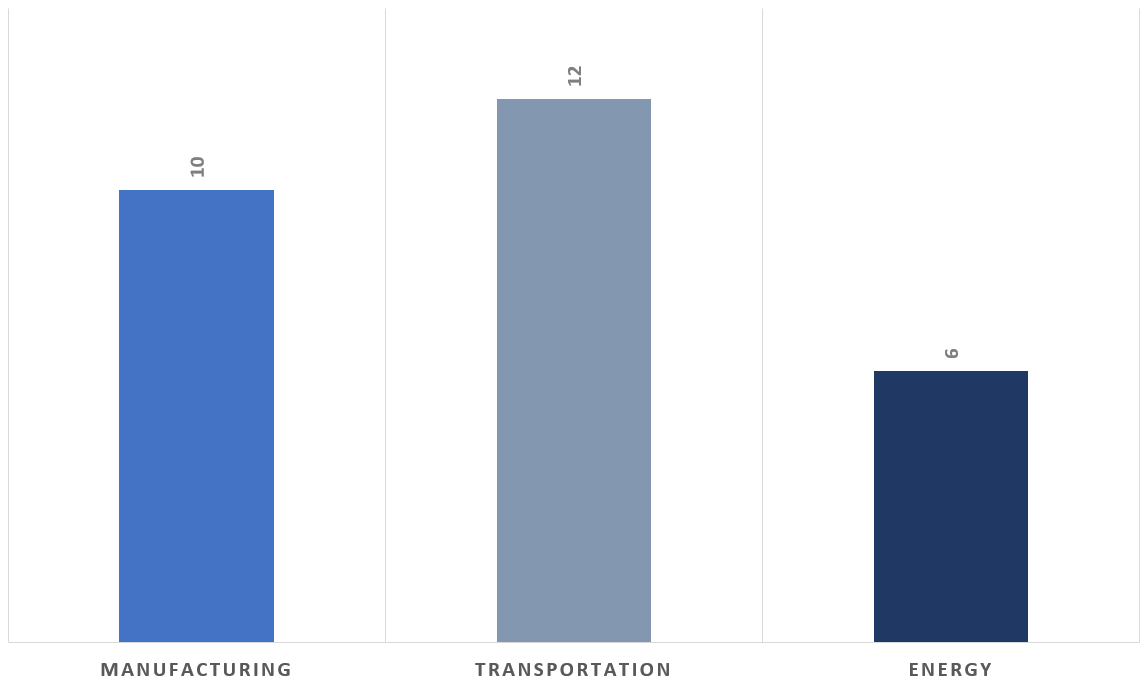
Distribution of IIoT-PdM implementations across industrial sectors based on the studies reviewed in Section 5.2 (n = 28).

As shown in Figure 9, IIoT-based predictive maintenance implementations are most frequently reported in transportation (42.9%), followed by manufacturing (35.7%) and energy (21.4%) sectors. This distribution reflects the varying technological requirements, regulatory environments, and economic drivers across industries. Transportation’s prominence may be attributed to the high value of mobile assets and safety considerations, while manufacturing benefits from controlled environments conducive to IIoT deployment. Energy applications, though fewer in number, often involve large-scale, remote assets requiring specialized communication and monitoring solutions.

This section explores the diverse applications of IIoT in PdM across these three key industrial sectors, examining implementation architectures, sensor technologies, communication protocols, and domain-specific challenges.

#### 5.2.1. Smart Manufacturing

In smart manufacturing, IIoT-driven predictive maintenance transforms traditional factories into highly efficient, data-driven environments. By equipping machines with IIoT sensors, manufacturers can continuously monitor critical parameters such as temperature, vibration, and energy consumption. This data is then processed in real time, allowing for early fault detection and optimized maintenance schedules. This section discusses how IIoT supports PdM in smart manufacturing, with a focus on real-world implementations and case studies.

AK Kalusivalingam et al. proposed an IIoT architecture for predictive maintenance that begins with data collection from temperature, vibration, acoustic, and pressure sensors installed on critical machinery. The captured data is transmitted in real-time via wireless networks (Wi-Fi/Zigbee) to a central IoT platform at predefined intervals (every 5 s). A Raspberry Pi, equipped with AI modules, processes the data locally before forwarding it to a cloud platform (AWS IoT Core/Google Cloud) for advanced analysis. The system ensures data security through timestamping, encryption, and the implementation of TLS anc Secure Sockets Layer (SSL) protocols, adhering to data privacy regulations such as General Data Protection Regulation (GDPR). The architecture was tested in a controlled test bed to validate its effectiveness in predictive maintenance applications [114]. Also, Lahis Almeida et al. proposed an IIoT-based predictive maintenance system that collects data from vibration, temperature, and current sensors to monitor industrial machines. The system employs a Wi-Fi Mesh network for communication, ensuring seamless data transmission from the sensors to the processing unit. An ESP32-WROOM-32UE serves as the central processing unit, interfacing with industrial machines via MODBUS RTU and other protocols. The collected data is analyzed at the edge to detect anomalies, while a cloud-based dashboard provides real-time machine condition monitoring [115]. Eyup Cinar et al. proposed a predictive maintenance system utilizing an IIoT-based CPS architecture for industrial manufacturing. The system collects data from vibration, current, and torque sensors to monitor the condition of electric motors and autonomous transfer vehicles. MQTT is employed as the primary communication protocol, enabling real-time data transmission from sensors to a centralized monitoring platform. A cloud-based infrastructure supports data storage and processing, while statistical process control (SPC) techniques and deep learning models are used for anomaly detection and predictive analytics [116]. Ovidiu Vermesan et al. proposed an IIoT-based predictive maintenance system for industrial manufacturing, integrating multiple sensors such as vibration, temperature, sound, and current/voltage sensors. Data is collected wirelessly using Bluetooth Low Energy (BLE), LoRaWAN, and Wi-Fi, with multiple gateways aggregating the data to an edge computing unit. The system utilizes embedded AI algorithms running on an ARM Cortex-M4 microcontroller to process the data at the edge, allowing for real-time fault detection and predictive maintenance. This implementation enhances machine monitoring, reduces maintenance costs, and improves overall production efficiency in manufacturing environments [117].Riccardo Rosati et al. proposed an IoT-enabled predictive maintenance system that integrates condition monitoring data from various sensors, including vibration, current, and temperature sensors. The system employs MQTT for real-time data collection and transmission, ensuring efficient and scalable communication between industrial machines and a cloud-based Decision Support System (DSS). The cloud architecture, implemented using Microsoft Azure, provides predictive analytics and storage, allowing for continuous monitoring and real-time alerts. The system enables manufacturers to optimize maintenance schedules, reduce service costs, and improve productivity by leveraging machine learning models for anomaly detection and predictive insights [118]. Van Tung Tran et al. proposed an IIoT-based predictive maintenance framework for a laser plastic welding machine, integrating sensor data acquisition, edge computing, and machine learning. The system collects data from load cells, pressure sensors, and machine power/voltage readings, which are synchronized and transmitted via the MQTT protocol. An IoT edge device functions as a data acquisition unit and a time synchronization module to ensure accurate condition monitoring. The collected data is sent to an on-premise server, where it undergoes preprocessing, feature extraction, fault detection, and predictive analytics using machine learning models. A web-based dashboard allows for real-time monitoring and visualization of machine conditions, supporting predictive maintenance decisions [119]. Ashraf Aboshosha et al. proposed an IIoT-based predictive maintenance system for a corrugated cardboard production factory, integrating temperature, current, pressure, vibration, and gap sensors to monitor machine conditions in real time. The system employs the MQTT protocol for data transmission, enabling seamless communication between factory equipment and a cloud-based analytics platform. A centralized IoT gateway processes sensor data before forwarding it to the cloud, where predictive models analyze operational trends [120]. Viktor Artiushenko et al. proposed an IIoT-based tool condition monitoring (TCM) system for CNC machines, utilizing pressure sensors and a Raspberry Pi 4 Model B as the central processing unit. Sensor data is acquired via the Serial Peripheral Interface (SPI) bus and stored in Comma-Separated Values (CSV) files for further analysis. Experimental validation on a 3-axis CNC milling machine demonstrated the system’s ability to capture sensor data effectively at a 1 kHz sampling rate, providing valuable insights for predictive maintenance [121]. Also, Sarvesh Sundaram and Abe Zeid proposed a Smart Prognostics and Health Management (SPHM) approach am applied it in CNC machine. Their setup integrates acoustic emission sensors, vibration sensors, and current sensors, with signals processed through a custom-made RMS meter. The processed signals are then transmitted via a UMK-SE 11,25 cable to an MIO-16 data acquisition board, ensuring accurate measurement and analysis [122].Continuing with CNC machine monitoring, Al-Naggar et al. developed an IIoT-based system for real-time condition monitoring of four CNC machines. Their approach employs a Raspberry Pi 3 Model B+ as the processing unit, collecting real-time sensor data from vibration sensors mounted on the machines. The Raspberry Pi, configured with the IP address of the workstation router, transmits the acquired data to a centralized database via a wired connection [123].

As summarized in Table 15, various architectures for IIoT-based predictive maintenance in smart manufacturing demonstrate a common pattern of integrating diverse sensors, robust communication protocols, edge or cloud processing units, and analytical models to enable real-time monitoring and fault prediction across different industrial use cases.

#### 5.2.2. Transportation

In the transportation sector, IIoT-driven predictive maintenance is transforming how vehicles, fleets, and infrastructure are monitored and maintained. By integrating advanced sensors, onboard diagnostics (OBD-II), and wireless communication technologies, transportation systems can detect early signs of wear and faults in engines, braking systems, and other critical components. Real-time data transmission to cloud-based analytics platforms enables proactive maintenance, reducing downtime and enhancing safety. This section explores the role of IIoT in predictive maintenance across various transportation applications, highlighting real-world implementations and case studies, as summarized and compared in Table 16.

Priit Kullerkupp, proposed an IIoT-based predictive maintenance system for vehicles, leveraging OBD-II sensor data and Narrowband Internet of Things (NB-IoT) communication for real-time diagnostics. The system collects data from engine coolant temperature, fuel trim, mass airflow, and throttle position sensors, which are processed by a Raspberry Pi Zero WH connected to the vehicle’s onboard diagnostics system. Data transmission is handled via NB-IoT and MQTT, ensuring efficient, low-power communication with a cloud-based predictive maintenance platform. The collected data is analyzed in the cloud, where ThingsBoard provides real-time monitoring and visualization, enabling early fault detection and optimized maintenance scheduling [124]. Also, Chen et al. proposed an IIoT-based predictive maintenance framework for the automotive industry, leveraging real-time telematics data to optimize vehicle servicing and reduce unexpected failures. The system integrates sensors monitoring speed, vibration, and engine load, interfacing with the onboard diagnostic system (OBD-II) and electronic control units (ECUs). Data is transmitted via 5G networks, ensuring low-latency communication with cloud-based analytics platforms, where historical maintenance data is processed alongside external factors such as weather and traffic conditions. This approach enhances fleet management by enabling proactive maintenance strategies, improving vehicle reliability, and minimizing downtime [125]. Jorge Fernandes et al. proposed an IIoT-based predictive maintenance approach for the automotive industry, integrating condition-based maintenance (CBM) and PdM strategies. The study highlights the role of IoT sensors in continuously monitoring critical machine parameters, such as temperature and pressure, in a Renault Cacia manufacturing plant. Data is collected from industrial sensors and transmitted via corporate networks to a centralized monitoring system called “Smart Observer.” This system processes real-time sensor data, providing early failure detection and enabling proactive maintenance interventions. By leveraging digital dashboards and automated alerts, Renault Cacia successfully minimized machine downtime and optimized production efficiency [126]. Patrick Strauß et al. proposed an IIoT-based predictive maintenance system for brownfield industrial environments, retrofitting legacy machinery with low-cost sensors. The system collects data from temperature, vibration, and current sensors installed on heavy-lift electric monorail systems at BMW Group. For communication, a 5 GHz Wi-Fi connection is used to ensure reliable wireless data transmission. A Banana Pi serves as the processing unit, acquiring sensor data and transmitting it to a cloud-based Microsoft Azure IoT platform. The system enables real-time condition monitoring and integrates machine learning models for anomaly detection and predictive analytics, optimizing maintenance strategies and reducing unplanned downtime [127]. Rajesh P. K. et al. proposed a Digital Twin-based predictive maintenance system for automotive brake pads, integrating IoT sensors with real-time virtual modeling. The system monitors brake pressure at different vehicle speeds, transmitting data to the ThingWorx IoT platform for analysis. A digital twin model, developed using CREO Simulate, processes real-time sensor inputs to predict brake pad wear and optimize maintenance schedules. This approach enhances vehicle safety and efficiency by enabling continuous condition monitoring and simulation-driven predictive analytics [128]. Iron Tessaro et al. proposed an IIoT-based predictive maintenance system for automotive engine components using real-time data acquisition. The system collects data from pressure and temperature sensors installed in turbocharged petrol engines. Data transmission is handled through ECU-based logging and remote storage, ensuring continuous monitoring of engine conditions. This approach enables early fault detection and optimized maintenance scheduling, improving engine reliability [129]. Furthermore, Vaibhav Mittal et al. proposed an IoT-enabled predictive maintenance system for sustainable transportation fleets, retrofitting electric buses, hybrid cars, electric trucks, and CNG-powered vans with real-time monitoring capabilities. The system collects data from IoT sensors measuring engine temperature, battery voltage, and brake wear percentages. Sensor data is transmitted wirelessly to a centralized cloud-based analytics platform, where machine learning algorithms analyze the input to detect anomalies and predict failures. The architecture supports real-time condition monitoring, reduces downtime, and optimizes maintenance strategies. Validation through live fleet data and historical comparisons confirmed the system’s adaptability and impact on sustainability and cost reduction. Data security and ethical considerations, including encryption and consent-based data handling, are integrated to ensure responsible deployment [130]. Lavish Kansal et al. proposed an IoT-enabled predictive maintenance system designed for sustainable transportation fleets, focusing on commercial trucks and public transport vehicles. Their system collects real-time data from sensors that monitor critical vehicle parameters, including engine temperature, battery voltage, tire pressure, brake pad thickness, and oil levels. The data is wirelessly transmitted to a cloud-based platform for centralized analysis, where maintenance schedules are dynamically adjusted based on real-time conditions and wear patterns. This proactive approach ensures timely maintenance interventions, improving fleet reliability, extending component lifespan, and promoting more efficient and environmentally friendly maintenance strategies [131]. Rohit Dhall and Vijender Solanki proposed an IoT-based predictive maintenance system for connected cars, replacing traditional periodic maintenance schedules with cloud-based analytics and real-time data collection. Their system integrates various in-vehicle sensors to monitor parameters such as fuel level, coolant temperature, engine oil status, and mileage. Data is transmitted through MQTT, with vehicles sending sensor data to IoT gateways like Eclipse Kura, which forward the information to cloud-hosted brokers. Subscribers receive this data, which is then processed by downstream services such as Apache Kafka, Storm, or Hadoop for analytics. The platform enables real-time fault alerts, maintenance scheduling via workflow automation, and predictive analytics dashboards for continuous monitoring. Simulations using MQTT client utilities demonstrated the feasibility of this architecture in generating actionable insights and reducing unnecessary maintenance costs. Cost analysis showed a potential 30 percent reduction in maintenance expenses and significant savings in outage costs for fleet operations, confirming the system’s economic benefits and operational efficiency [132]. Amitabh Bhargava et al. proposed an IIoT-integrated framework for predictive maintenance in vehicular logistics systems, combining artificial intelligence, cloud computing, and real-time sensor monitoring. The system, named IoT-ILTMF (IoT-assisted Interconnected Logistics Transportation and Management Framework), is designed to collect and analyze data from multiple vehicle-mounted sensors that monitor parameters such as engine temperature, fuel level, axle vibrations, GPS location, and vehicle speed. This sensor data is transmitted wirelessly to a cloud computing platform, where AI algorithms detect abnormalities and trigger predictive maintenance protocols. The framework also guides semi-autonomous vehicles with contextual data, including nearby service stations and optimal delivery routes, to minimize delays. Compared to other models, IoT-ILTMF demonstrated significant improvements in performance, energy efficiency, and cost reduction, especially at scale. Real-world simulations with varying fleet sizes showed performance improvements from 77 percent to 98 percent, increased customer satisfaction by up to 80 percent, and reduced operational costs and energy consumption through intelligent automation and optimized route planning. The system leverages emerging technologies such as 6G for low-latency data transmission and blockchain for enhanced security, ensuring robust and scalable implementation across transportation networks [133].

Mohamed Amine Ben Ali et al. proposed an advanced IIoT architecture for predictive maintenance in green transportation systems, integrating sensor networks, edge computing, fog computing, and cloud analytics. Vehicles, including EVs and hydrogen-powered units, were equipped with automotive-grade sensors (e.g., temperature, vibration, and energy consumption) connected via CAN-FD for critical signals and low-power mesh networks for auxiliary data. Sensor data was processed by ruggedized edge computers with ARM Cortex-A76 CPUs and TPUs running real-time OS and containerized microservices for deterministic event handling and on-board inference. Communication with infrastructure used IEEE 802.11p in depots and LTE-M/NB-IoT on routes. Edge nodes maintained local time-series databases, while depot-level fog clusters (with NVIDIA T4 GPUs) hosted digital twins and exposed standardized APIs. The cloud tier, deployed on hyperscale platforms, supported a data lake for long-term analytics, enabling secure, scalable PdM across green transit fleets [134].

Sheraz Aslam et al. developed a hierarchical IIoT architecture for predictive maintenance in smart port environments, targeting container handling equipment (CHE) such as straddle carriers. The system integrates hydraulic pressure sensors and programmable logic controllers (PLCs) to monitor machine conditions in real time. Sensor data is transmitted to far-edge gateways responsible for initial preprocessing, including signal smoothing and noise filtering. These gateways interface with cloud infrastructure over secure wireless channels, enabling seamless transmission of structured datasets. The cloud platform hosts multiple machine learning models, including ANN, RF, DT, XGBoost, and GNB, within a modular analytics framework to detect anomalies such as inverter overheating. The architecture supports edge-cloud collaboration, ensuring low-latency monitoring and centralized analytics across port-scale deployments. It also enables flexible model deployment and scalable integration with port management systems [135].

#### 5.2.3. Energy

In the energy sector, IIoT is redefining maintenance operations by enabling continuous oversight of essential infrastructure. Equipped with smart sensors and connected systems, components like transformers, turbines, and grid controllers can be closely monitored for performance deviations and early indicators of failure. The constant flow of operational data to intelligent analytics platforms allows for timely interventions, minimizing disruptions, and extending asset life. This section examines the growing impact of IIoT-enabled predictive maintenance in energy environments, showcasing practical applications and real-world benefits, as detailed in the comparative overview provided in Table 17.

Starting with Federico Civerchia et al., who developed the NGS-PlantOne system, an IIoT-based predictive maintenance solution designed for industrial machinery monitoring. The system uses battery-powered sensor nodes to collect temperature and vibration data, communicating via 6LoWPAN, RPL, and CoAP protocols. Data is transmitted through multi-interface gateways, which bridge the sensor network with a centralized Remote Control and Service Room (RCSR) for analysis and visualization. Deployed in a power plant across three operational areas, the system included 33 sensors and demonstrated reliable performance with communication latency under 330 ms and projected battery life of up to 150 days. The architecture supports plug-and-play scalability, energy efficiency, and real-time fault detection, enabling timely and cost-effective maintenance interventions [136]. Radhakrishnan et al. proposed an intelligent control system for wind turbine farms, leveraging IoT sensors and machine learning to enable predictive maintenance and real-time optimization. The system collects data such as wind speed, direction, temperature, and humidity using distributed IoT devices installed on turbines. This data is processed via cloud-based machine learning algorithms to predict power output, detect faults, and forecast wind conditions. A control algorithm adjusts turbine pitch angle and rotor speed accordingly, implemented on microcontrollers integrated with the IoT nodes. This approach enhances turbine efficiency, reduces maintenance costs, and supports scalable, autonomous control across wind farms [137]. Lei Gong and Yanhui Chen proposed an IoT-based predictive maintenance system for wind turbines, using sensors to collect temperature, vibration, and rotational speed data from turbine components. These readings are aggregated by microcontroller units and transmitted via gateways using Wi-Fi and GSM protocols to cloud infrastructure for analysis. The architecture supports continuous monitoring and early fault detection, enabling efficient scheduling of maintenance activities. By ensuring seamless data flow from sensor layer to cloud, the system enhances operational visibility and turbine reliability while reducing downtime and maintenance costs [138]. Also, Mindaugas Jankauskas et al. proposed an IoT-enabled predictive maintenance system for wind turbines, utilizing real-time data collected from SCADA-integrated sensors monitoring key operational parameters such as gearbox temperature, wind speed, ambient temperature, and generator and rotor RPM. These IoT sensors feed continuous data streams into a centralized analytics platform where predictive models analyze deviations to detect anomalies. The system enables early fault detection up to 37 days in advance, reducing manual inspections and supporting timely, condition-based maintenance to improve reliability and minimize downtime [139]. Bramantyo et al. proposed an IoT-based monitoring architecture for oil and gas refineries, aimed at enhancing real-time visibility of critical components such as pressure relief valves and steam traps. The system utilizes differential pressure and acoustic sensors connected through edge devices and local area networks, enabling continuous data acquisition. Data is transmitted to cloud-based dashboards via embedded networking connectors, supporting remote monitoring and timely alerts. Communication between IoT devices, PLCs, and SCADA systems is facilitated using protocols like MQTT, ensuring seamless integration and low-latency performance. This approach enables condition-based maintenance, improves operational efficiency, and supports scalable deployment across refinery environments [140]. Tiantian Xu et al. introduced an IIoT-enabled digital twin framework for intelligent operation and maintenance of wind turbine gearboxes. The system fuses multi-source sensor data including temperature, pressure, power, and vibration collected via SCADA-integrated IIoT nodes deployed on turbine components. These data streams are transmitted in real-time to cloud-based digital twin environments, where virtual replicas of the gearboxes continuously update and mirror physical behavior. This integration supports condition monitoring, anomaly detection, and remote visualization, enabling timely maintenance planning and improved reliability of wind turbine gear systems [141].

#### 5.2.4. Discussion and Comparative Analysis of IIoT Applications in PdM

The analysis of IIoT-based predictive maintenance implementations across smart manufacturing, transportation, and energy sectors reveals distinct architectural patterns, deployment challenges, and performance trade-offs shaped by sector-specific requirements.

Sector-Specific Architectural Patterns:

In smart manufacturing, IIoT-PdM systems prioritize high-frequency multi-sensor monitoring (vibration, temperature, current) with strong reliance on edge computing for real-time responsiveness. As shown in Table 15, deployments commonly employ Raspberry Pi or industrial gateways for local processing, coupled with cloud platforms (AWS, Azure) for long-term analytics and model retraining [114,118]. The predominance of MQTT as a communication protocol reflects the need for lightweight, scalable messaging in noisy industrial environments. However, implementations vary significantly in their edge-cloud partitioning—some systems perform anomaly detection entirely at the edge [117], while others rely on cloud-based decision support [118]. This variation highlights the ongoing tension between latency requirements and computational capabilities in factory settings.

Transportation applications demonstrate greater heterogeneity, ranging from vehicle-level diagnostics using OBD-II interfaces [124] to fleet-wide predictive analytics integrating telematics, weather, and traffic data [125]. As summarized in Table 16, communication technologies span from low-power NB-IoT for remote monitoring to high-bandwidth 5G for real-time video and sensor streams. A notable trend is the emergence of digital twin implementations for critical components like brakes and engines [128], enabling simulation-driven maintenance planning. The transportation sector also shows the most advanced integration of sustainability considerations, with several studies explicitly optimizing maintenance schedules for energy efficiency and emissions reduction [130,134].

Energy sector implementations, particularly in wind turbine monitoring, reveal the most mature convergence of IIoT with legacy supervisory systems (SCADA). As indicated in Table 17, these systems emphasize long-range, low-power communication (LoRaWAN, 6LoWPAN) for distributed assets [136], often combining edge preprocessing with cloud-based digital twins for comprehensive condition monitoring [141]. The energy sector also demonstrates the longest prediction horizons, with some systems detecting faults up to 37 days in advance [139], enabled by extensive historical data from SCADA systems.

Common Challenges and Trade-offs:

- Communication vs. Power Constraints: Systems requiring real-time monitoring (e.g., manufacturing robotics) favor high-bandwidth Wi-Fi/5G, while remote asset monitoring (e.g., wind farms, pipelines) prioritizes low-power, long-range protocols like LoRaWAN at the expense of latency and data rate.
- Edge vs. Cloud Processing Balance: While edge computing reduces latency and bandwidth usage, it faces limitations in model complexity and retraining capabilities. The optimal partitioning varies by application: time-critical anomaly detection at the edge versus resource-intensive model training and fleet optimization in the cloud.
- Brownfield Integration Complexity: Retrofitting legacy equipment with IIoT sensors presents significant challenges in sensor placement, power supply, and protocol translation [127]. Successful implementations often employ gateway-based architectures that bridge legacy protocols (MODBUS, PROFINET) with modern IoT standards.
- Data Heterogeneity and Integration: The diversity of sensor types, sampling rates, and communication protocols across different equipment and vendors complicates the development of unified IIoT platforms for PdM, particularly in multi-vendor industrial environments.
- Security and Privacy Considerations: While several studies mention encryption and secure protocols [114,130], comprehensive security architectures addressing the expanded attack surface of IIoT systems remain underdeveloped in the reviewed implementations.

Performance Gaps and Research Opportunities:

Despite significant advances, several performance gaps persist:

- Limited reporting of end-to-end latency beyond communication delays, omitting processing times at edge and cloud layers.
- Inconsistent evaluation metrics across studies, with some focusing solely on prediction accuracy while others emphasize system availability or maintenance cost reduction.
- Scarce real-world longitudinal validation of deployed systems, with most evaluations based on controlled testbeds or historical data rather than operational environments.
- Under-explored human factors in IIoT-PdM systems, particularly regarding operator interfaces, trust in automated recommendations, and skill requirements for system maintenance.

These findings underscore that successful IIoT-PdM implementation requires careful alignment of architectural choices with sector-specific requirements, operational constraints, and existing infrastructure. The transition from isolated IIoT deployments to integrated AIoT ecosystems, where intelligence is seamlessly distributed across edge and cloud resources, represents the next evolutionary step, as explored in the next section.

### 5.3. AIoT-Driven Approaches for Predictive Maintenance

The integration of Artificial Intelligence with the Industrial Internet of Things, collectively known as Artificial Intelligence of Things, has given rise to a new generation of intelligent, distributed systems for Predictive Maintenance. While existing literature often treats AI and IIoT as separate contributors to maintenance, AIoT embodies a deeper, integrated framework where data acquisition, intelligent analysis, and system actuation are seamlessly connected. This integration has transformed PdM from static, threshold-based monitoring into a dynamic, context-aware, and adaptive process aligned with the objectives of Industry 5.0, human-centric, resilient, and sustainable operations. In this section, we examine the role of AIoT in enabling and advancing PdM. We organize our discussion into two main categories: (i) Evolving Predictive Maintenance Methodologies with AIoT, which demonstrate how AIoT augments maintenance paradigms, and (ii) Next-Generation AIoT Solutions for Predictive Maintenance, which reflect novel, intelligent systems and approaches that have emerged from this convergence.

#### 5.3.1. Evolving Predictive Maintenance Methodologies with AIoT

This subsection highlights how AIoT reinforces and extends well-established maintenance strategies such as Condition-Based Maintenance, Prognostics and Health Management, hybrid approaches, and prescriptive maintenance. These systems offer increased accuracy, responsiveness, and automation in decision-making across industrial assets.

##### Condition-Based Maintenance Enabled by AIoT

Condition-Based Maintenance has undergone a transformative shift through the integration of AIoT, evolving from rigid threshold-based systems and manual inspections to intelligent, adaptive frameworks powered by real-time sensor data and edge AI. AIoT enables continuous learning from operational data, dynamic thresholding, and early anomaly detection, exemplified by systems in manufacturing that identify subtle changes to trigger preemptive actions. Rosati et al. demonstrated this in smart manufacturing using MQTT to transmit sensor data to edge devices. These devices employ machine learning to detect behavioral anomalies, enabling adaptive thresholds and early fault detection. The system further incorporates a cloud-based Decision Support System for centralized analysis, balancing edge responsiveness with cloud intelligence [142]. Kabashkin and Shoshin introduced a three-tiered AIoT architecture for aircraft health monitoring that integrates satellite sensors, 6G connectivity, and edge ML to improve predictive accuracy and cost-efficiency [143]. In the automotive sector, Kalalas et al. employed edge service migration and AI models in a 5G-enabled system that achieved 15.1 ms inference time with high fault recall [144]. He et al. optimized CBM in wind turbines using IoT data and AI-driven RUL predictions to reduce economic loss and inspection costs [145]. Valjus and Säll proposed a human-centric CBM framework for LNG infrastructure that integrates conversational AI agents for interactive decision-making [146], while Nguyen used a CNN-GRU model for real-time firefighting pump monitoring, achieving 98.17% accuracy [147]. Bin-Habtoor and Fatima applied decision trees and neural networks to ESP systems, optimizing model selection via MOORA to maximize uptime [148]. Finally, Marti-Puig et al. used ELM-based models on IoT-monitored wood-residue systems, enabling effective anomaly detection without hardware changes [149]. Collectively, these advancements exemplify how AIoT elevates CBM into a proactive, scalable, and domain-agnostic strategy that enhances reliability and operational efficiency across industries.

Table 18 summarizes recent AIoT-based CBM approaches, highlighting their domains, techniques, architectures, and limitations.

##### AIoT-Driven Prognostics and Health Management

Prognostics and Health Management is undergoing a paradigm shift with the integration of AIoT, moving beyond traditional predictive maintenance by offering intelligent, real-time insights into system degradation and failure evolution. The integration of AIoT into PHM frameworks has substantially enhanced this capability, enabling real-time, intelligent, and predictive insights into system health across diverse industrial settings. Mazurkiewicz et al. [150] propose an AIoT-based PHM approach that integrates reliability-centered maintenance with process optimization, using multisensor IoT data and AI models to classify health states, predict Remaining Useful Life, and enhance operational efficiency through adaptive decision-making. Ramos et al. [151] introduce a machine learning-based pipeline for PHM in petrochemical systems, combining semi-supervised CNNs for anomaly detection, XGBoost for RUL prediction, and SHAP for model explainability, achieving robust early fault detection and high predictive accuracy.Similarly, Kumar and Kota [152] present an IoT-enabled SHM framework using triaxial accelerometers and AI-based vibration analysis to localize structural damage, supported by web and mobile interfaces for real-time visualization, exemplifying a full-stack AIoT solution. In manufacturing, Wang et al. [153] enhance PHM with a PSO-CNN and GRU-Attention model for RUL prediction, integrating Augmented Reality to guide maintenance, achieving 98.16% accuracy and reducing downtime. In aviation, Kabashkin [154] integrates Foundation Models and Federated Learning within an AIoT-based aircraft PHM framework, ensuring edge-level anomaly detection, data privacy, and global model convergence, thereby improving diagnostic precision and scalability. Meanwhile, Yousuf et al. [155] develop a cost-effective AIoT solution for industrial motor monitoring using sensor data transmitted via GSM to the cloud, facilitating remote fault detection and real-time alerts through rule-based logic, successfully reducing unplanned downtimes in real-world deployments. Collectively, these methodologies demonstrate how AIoT reinforces PHM by fusing AI-driven analytics with ubiquitous sensing and communication, delivering scalable, adaptive, and explainable maintenance solutions.

Table 19 provides a comparative overview of AIoT-enabled PHM approaches, focusing on the applied AI techniques, IoT/edge components, key outcomes, and identified limitations.

##### AIoT-Driven Prescriptive Maintenance

Prescriptive maintenance refers to the most advanced form of maintenance strategy that not only predicts when a failure might occur but also recommends optimal actions to prevent it, balancing cost, downtime, and operational efficiency. The integration of Artificial Intelligence of Things enhanced prescriptive maintenance by enabling intelligent, autonomous decision-making at the edge or in distributed systems. This evolution is reflected in several recent studies. Gibey et al. [156] propose a strategy for PEMFC systems that integrates real-time IoT data with BiESN and BiLSTM models for fault diagnosis and Remaining Useful Life estimation. Papaioannou et al. [157] introduce LP-OPTIMA, a lightweight AIoT framework for low-power devices that enables local anomaly detection and prescriptive decisions without reliance on cloud infrastructure. Zhao et al. [158] present TranDRL, which combines Transformer-based RUL prediction with deep reinforcement learning and RLHF to optimize maintenance in federated industrial settings. Ordieres-Meré et al. [159] deploy a petrochemical prescriptive system using sensor data and DRL for adaptive, automated decision-making. Meanwhile, Petroutsatou et al. [160] develop PREMSYS, a system that leverages real-time and historical data to suggest cost-effective and sustainable maintenance actions for construction equipment. These works illustrate how AIoT is driving the transition from reactive and predictive models to fully autonomous, context-aware maintenance ecosystems in line with Industry 4.0 and 5.0 paradigms.

Table 20 presents a comparative analysis of recent AIoT-driven prescriptive maintenance studies, highlighting the employed AI techniques, the role of IoT, key prescriptive capabilities, and their respective limitations.

##### Hybrid AIoT-Based Maintenance Strategies

Hybrid AIoT-based maintenance strategies merge the strengths of physics-based models with data-driven AI approaches to enhance the accuracy and adaptability of predictive maintenance systems. Rosati et al. [118] propose a hybrid decision support system that integrates IoT-enabled multi-level data acquisition with a Random Forest-based machine learning layer, allowing for indirect prediction of equipment degradation through process quality indicators. The system achieves high predictive performance (R^2^ = 0.868, MAE = 0.089) without relying on run-to-failure data and supports cloud-based incremental learning for real-time updates. Nascimento and Viana [161] extend this concept by employing physics-informed recurrent neural networks (PI-RNNs) that combine Paris’ law for crack propagation with fleet-level IoT operational data to predict cumulative fatigue damage and remaining useful life, even when only a subset of equipment has observed damage data. Similarly, Yucesan and Viana [162] introduce a hybrid PINN model for wind turbine maintenance, incorporating grease degradation patterns and SCADA-like sensor data into a recurrent neural network to optimize regreasing schedules based on both lubricant condition and fatigue prognosis. Aboshosha et al. [120] develop a two-stage architecture that integrates fuzzy logic and artificial neural networks, using IoT-acquired sensor and operator data to detect faults and predict remaining useful life. This combination of interpretability and deep learning enables effective maintenance decisions in industrial environments. Traini et al. [163] address tool wear monitoring in milling by combining initial physics-based models with progressively integrated data-driven predictions as IoT sensor data becomes available, enabling accurate condition monitoring throughout the tool’s lifecycle. These hybrid AIoT frameworks collectively demonstrate enhanced reliability, adaptability, and efficiency in predictive maintenance, especially in complex or data-scarce industrial contexts.

Table 21 presents a comparative overview of recent studies on hybrid AIoT-based maintenance strategies, highlighting their architectural approaches, AI techniques, IoT integration, key features, and identified limitations.

#### 5.3.2. Next-Generation AIoT Solutions for Predictive Maintenance

Beyond enhancing existing methodologies, AIoT has unlocked entirely new paradigms in predictive maintenance. This section explores emerging innovations, including edge–cloud collaborative intelligence, self-adaptive learning systems, and digital twins, each contributing to the development of more autonomous, context-aware, and efficient maintenance ecosystems.

##### Edge–Cloud Collaborative Intelligence

AIoT systems increasingly leverage collaborative intelligence architectures that distribute computational tasks between edge devices and the cloud. To illustrate the role of Edge–Cloud Collaborative Intelligence in PdM, several studies have proposed frameworks that dynamically allocate AI processing across the edge–cloud continuum to optimize responsiveness, reliability, and scalability in Industrial IoT environments.Xu et al. present a layered architecture where Deep Reinforcement Learning policy networks operate on edge devices for low-latency control, while the cloud oversees model training, global coordination, and dynamic resource management. Their Markov Decision Process-based system reduces latency and costs while enhancing anomaly detection, equipment uptime, and overall reliability [164]. Yuan et al. demonstrate energy-efficient, real-time monitoring by integrating a Deep Q-Network that schedules processing tasks between IoT sensors, edge nodes, and the cloud, ensuring accurate fault prediction even in bandwidth-constrained environments [165]. Sathupadi et al. employ lightweight KNN models at the edge for immediate anomaly detection, while LSTM models in the cloud handle long-term failure prediction, achieving 35% lower latency, 28% reduced energy consumption, and 60% bandwidth savings [166]. Kota introduces a robust Edge–Cloud PdM framework where edge devices process up to 850 GB/day for anomaly detection and RUL estimation, maintaining autonomous operations for 72 h and cutting cloud compute costs by 31% while achieving 92% prediction accuracy and a 23% increase in Overall Equipment Effectiveness [167]. Bemani proposes a federated learning-based setup where edge nodes conduct local model training for RUL and anomaly detection, while the cloud performs global aggregation using over-the-air techniques and robust gradient descent, enabling spectrum-efficient learning and low-latency responses across heterogeneous environments [168]. In the pharmaceutical sector, Parapalli and Shetty design a hybrid Edge–Cloud architecture embedded within Manufacturing Execution Systems, enabling predictive analytics through local anomaly detection and cloud-based AI optimization, resulting in 50% fewer unplanned downtimes and 75% fewer equipment breakdowns [169]. Mehta and Verma’s CollabRULe framework optimizes edge server placement via a QoS-aware model and uses federated learning for decentralized RUL prediction, achieving a 60% reduction in network energy usage and a 35% latency decrease while maintaining high prediction accuracy [170]. Lastly, Jia and Ren introduce IntelliPdM, which uses lightweight edge models for sub-50 ms anomaly detection and cloud-based deep learning, synthetic data fusion, and global analytics, delivering 95–99% accuracy, a 10× ROI, and significant cost and breakdown reductions in real-world deployments [171]. Collectively, these works underscore how collaborative Edge–Cloud intelligence empowers AIoT-based PdM systems to meet the latency, scalability, and efficiency demands of Industry 5.0.

Table 22 provides a comparative summary of recent studies that implement Edge–Cloud collaborative intelligence architectures for predictive maintenance in AIoT systems, highlighting their AI techniques, IoT integration strategies, key outcomes, and limitations.

##### Self-Adaptive and Federated Learning

The synergy between Federated Learning (FL) and AIoT improves PdM by facilitating decentralized learning, model personalization, and adaptability to heterogeneous and evolving industrial environments. Several recent studies illustrate these advancements, with their key attributes compared in Table 23. Hosni et al. propose a secure and scalable FL framework for PdM in industrial systems that leverages encrypted model updates and a centralized aggregation mechanism to iteratively refine a global model, achieving 97.2% accuracy and reducing communication overhead by 40% [172]. Alshkeili et al. present an explainable and privacy-preserving FL approach that incorporates SHAP and LIME for transparency and supports continuous adaptation via IIoT-generated data, achieving 98.15% accuracy and outperforming traditional models [173]. Kabashkin et al. integrate Foundation Models with FL in aircraft systems, introducing self-adaptive mechanisms for adjusting training parameters based on resource availability, thereby enhancing prediction accuracy, reducing false alarms, and enabling digital twin capabilities [154]. Becker et al. utilize lightweight autoencoders and FedAvg in IIoT to monitor rotating machinery, where local models personalize to individual machine behavior and collaboratively learn generalized failure patterns without exposing raw data [174]. Liu et al. enhance FL with knowledge distillation and a self-adaptive loss balancing strategy, enabling soft target sharing and improved generalization in heterogeneous AIoT conditions, surpassing FedAvg in accuracy while reducing communication costs [175]. Alatawi et al. introduce SAFEL-IoT, which uses adaptive aggregation based on client divergence, integrates LSTM with explainability tools, and employs homomorphic encryption and differential privacy to achieve secure, low-latency, and interpretable anomaly detection in Industry 5.0 [176]. Collectively, these works exemplify how AIoT-powered self-adaptive and federated learning frameworks can meet the stringent demands of modern PdM by enabling secure, scalable, and intelligent distributed learning systems.

##### Digital Twin-Driven Predictive Maintenance

A Digital Twin (DT) is a dynamic, virtual representation of a physical system that continuously synchronizes with real-world data to enable monitoring, simulation, and decision-making. When enhanced with AIoT, the convergence of AI and Industrial IoT, Digital Twins evolve into intelligent systems capable of real-time diagnostics, failure prediction, and autonomous control.In this context, Khan et al. propose a data-driven DT framework for CNC-based smart manufacturing that uses IIoT sensors to capture cutting parameters and applies machine learning models like Random Forest and XGBoost to predict surface roughness and power consumption, integrating these predictions into a real-time DT for continuous optimization and maintenance planning [177]. Kerkeni et al. build a DT of a winding machine and integrate it with a hybrid AI model combining autoencoders and LSTMs, using IIoT-collected data over Profinet to detect anomalies with 98% accuracy in unlabeled datasets, enabling unsupervised fault prediction in real industrial settings [178]. Prasath presents a DT-based predictive maintenance system for smart grids where IIoT sensors feed condition data into AI models for early fault detection and proactive scheduling, demonstrating the transition from reactive to intelligent maintenance in energy systems [179]. Abdullahi et al. develop a distributed DT architecture for wind turbines, incorporating fog and cloud layers to process IIoT sensor data with lightweight ML models at the edge and more complex models in the cloud, resulting in scalable, low-latency predictive diagnostics [180]. Hamel et al. propose PMI-DT, a DT system built on Microsoft Azure that combines inspection data and fatigue testing to train decision tree classifiers with 100% accuracy, showing how AI and IoT can support virtual inspection and predictive maintenance in additive manufacturing [181]. Wang applies a DT system to oil and gas pipelines by fusing real-time IoT sensor data with physics-based simulation models and machine learning classifiers to identify leaks and corrosion, achieving robust performance through hybrid modeling and predictive insights [182]. Hu et al. introduce a dual-DT approach to optimize DNN inference in AIoT systems, using a Decision-Support DT for offloading strategy simulation and a Status-Estimator DT for progress prediction, thus enhancing predictive maintenance capabilities through adaptive Edge–Cloud collaboration [183]. Finally, Singh et al. design a DT for induction motors using Simulink and IIoT sensors, where embedded AI models accurately diagnose electrical faults and estimate RUL, proving the lightweight and effective nature of AIoT-driven DTs in Industry 4.0 settings [184]. Collectively, these works demonstrate how AIoT technologies empower Digital Twins to deliver real-time, data-driven, and proactive predictive maintenance across diverse industrial domains.

Table 24 presents a comparative analysis of recent studies that integrate AIoT with DT technologies for predictive maintenance. The comparison highlights the AI techniques, IoT strategies, DT functionalities, key contributions of each approach, and their respective limitations.

Together, the applications and innovations reviewed in this section illustrate how AIoT is reshaping predictive maintenance from reactive and periodic strategies to intelligent, adaptive, and autonomous processes. By fusing sensing, computation, and learning across physical and digital domains, AIoT not only augments traditional methodologies but also paves the way for new paradigms such as federated intelligence and digital replication. These developments position AIoT as a foundational enabler of next-generation maintenance systems in Industry 5.0.

#### 5.3.3. Discussion and Comparative Analysis of AIoT-Driven Approaches for PdM

The convergence of AI and IIoT within AIoT-driven predictive maintenance frameworks has catalyzed both the evolution of traditional maintenance methodologies and the emergence of novel architectural paradigms. This analysis synthesizes insights from the reviewed studies, highlighting comparative effectiveness, real-world applicability, and critical trade-offs.

Evolving Methodologies: From Enhancement to Autonomy

The reviewed AIoT-enhanced methodologies reveal a clear progression toward greater autonomy and contextual awareness. Condition-Based Maintenance (CBM) has transitioned from static threshold-based alerts to adaptive, learning-driven systems. As shown in Table 18, implementations range from cloud-centric decision support [142] to edge-optimized real-time inference [144]. However, a key trade-off emerges: systems achieving high accuracy and low latency (e.g., 15.1 ms inference time in automotive applications [144]) often rely on high-bandwidth connectivity (5G/6G) and substantial edge compute resources, limiting their deployment in resource-constrained or remote environments.

Prognostics and Health Management (PHM) benefits significantly from AIoT through improved data fusion and explainability. Systems integrating multi-sensor data with hybrid AI models (e.g., PSO-CNN with GRU-Attention [153]) achieve high diagnostic accuracy (98%) but at the cost of increased computational complexity. The incorporation of explainability tools like SHAP [151] addresses the critical need for trust and interpretability in safety-critical domains, though scalability to real-time streaming data remains a challenge.

Prescriptive Maintenance represents the frontier of autonomy, where AIoT systems not only predict failures but also recommend optimal interventions. Frameworks like TranDRL [158] demonstrate how Transformer-based RUL prediction combined with Deep Reinforcement Learning (DRL) can optimize maintenance policies in federated settings. However, as noted in Table 20, these advanced systems face significant limitations, including high communication overhead, model complexity, and dependency on high-quality, multi-source data.

Hybrid Strategies that combine physics-based models with data-driven AI offer a promising path for data-scarce or high-risk applications. Studies like those employing Physics-Informed Neural Networks (PINNs) [161,162] leverage domain knowledge to constrain AI predictions, improving generalization. Yet, their effectiveness is contingent on the accuracy of the underlying physical models and requires significant domain expertise for calibration.

Next-Generation Architectures: Balancing Intelligence and Practicality

Edge–Cloud Collaborative Intelligence architectures fundamentally address the latency-bandwidth-accuracy trilemma. As the comparative results in Table 22 indicate, effective task partitioning between edge and cloud can yield substantial benefits: 35% latency reduction, 60% bandwidth savings [166], and 31% lower cloud costs [167]. The choice of partitioning strategy, however, is highly application-dependent. Lightweight models (e.g., KNN) at the edge enable rapid anomaly detection but may sacrifice accuracy compared to cloud-based deep learning models.

Federated Learning (FL) and Self-Adaptive Learning frameworks address critical concerns of data privacy, personalization, and continuous adaptation. FL approaches, such as those employing encrypted updates and adaptive aggregation [172,176], maintain high accuracy (98%) while preserving data sovereignty. However, they introduce new challenges: communication overhead, vulnerability to adversarial attacks, and increased complexity in model synchronization and client management.

Digital Twin (DT) implementations demonstrate the power of virtual replication for simulation and planning. DTs range from component-level models for specific failure modes to system-wide simulations incorporating operational and environmental factors. Their utility is greatest in scenarios allowing for “what-if” analysis and proactive planning, such as optimizing maintenance schedules for wind turbines [180] or simulating pipeline integrity [182]. The primary barriers to broader DT adoption are the high cost of model development, the need for continuous high-fidelity data synchronization, and significant computational resources.

Comparative Effectiveness and Real-World Applicability

A critical synthesis reveals that no single AIoT approach is universally superior. The optimal strategy depends on a confluence of factors:

- Data Availability and Quality: Data-rich environments favor complex deep learning and prescriptive models, while data-scarce or noisy settings benefit from hybrid physics-AI or simpler edge-based anomaly detection.
- Latency and Connectivity Requirements: Time-critical applications in manufacturing necessitate edge-heavy or Edge–Cloud collaborative designs, whereas fleet or infrastructure management with less stringent latency needs can leverage cloud-centric analytics.
- Safety Criticality and Interpretability Needs: High-risk sectors (aviation, energy) prioritize explainable and reliable PHM systems, even at the expense of some predictive accuracy or model complexity.
- Infrastructure and Cost Constraints: Brownfield deployments must balance performance gains against integration complexity and cost, often favoring lightweight, retrofit-friendly solutions over transformative, AIoT-native architectures.

Remaining Gaps and Future Trajectory

Despite the advancements chronicled here, significant gaps impede widespread industrial adoption:

- Standardization and Interoperability: A lack of standardized interfaces, data formats, and evaluation metrics hinders the integration of diverse AIoT components and complicates comparative performance assessment.
- Lifecycle Management of AIoT Systems: The maintenance of the AIoT infrastructure itself—including model retraining, drift detection, software updates, and hardware lifecycle—is an underexplored but critical operational challenge.
- Human-AI Collaboration: Most research focuses on technical performance, with insufficient attention to human-in-the-loop interfaces, trust calibration, and the changing role of maintenance personnel in AIoT-augmented environments.
- Comprehensive Security Postures: While individual studies address encryption or secure aggregation, holistic security frameworks protecting the entire AIoT stack from sensor to cloud against evolving threats are still nascent.

In conclusion, AIoT-driven PdM represents a paradigm shift from isolated data collection and analysis to integrated, intelligent ecosystems. The transition from enhanced traditional methodologies to next-generation architectures underscores a move towards greater autonomy, adaptability, and alignment with Industry 5.0 values. Realizing the full potential of this convergence will require continued research not only into improving AI accuracy and IoT connectivity but also into overcoming the systemic challenges of integration, standardization, security, and human-centric design.

## 6. Discussion

AIoT is reshaping the landscape of PdM across various industrial sectors. This section synthesizes key insights from the reviewed literature, focusing on the advantages, concrete limitations, and future research directions of AIoT-driven PdM systems.

### 6.1. Advantages of AIoT in Predictive Maintenance

AIoT systems offer notable advantages across multiple dimensions:

- Performance and Accuracy Gains: Across the reviewed works, deep learning models such as CNN-LSTM [84], BiLSTM [92], and attention-based transformers like DSFormer [101] and STAR [103] achieve high predictive accuracy in Remaining Useful Life estimation and fault classification, often outperforming traditional methods in complex, multi-sensor environments.
- Real-time Decision Making at the Edge: Edge–Cloud collaborative architectures (e.g., [166,167]) enable low-latency inference while reducing communication and cloud dependency. Such frameworks offer tangible benefits like energy savings, improved equipment uptime, and higher Overall Equipment Effectiveness.
- Scalability via Federated and Self-Adaptive Learning: FL approaches [172,176] preserve data privacy while distributing learning across IIoT nodes. These architectures maintain high accuracy (up to 98.15%) and demonstrate resilience to communication failures and adversarial attacks.
- Domain Adaptability: AIoT solutions are successfully deployed across manufacturing, transportation, and energy sectors. For instance, [134,141] showcase robust, scalable implementations in EV transit and wind turbines, respectively.
- Hybrid Strategies for Generalization: Physics-informed models [161,162] and hybrid AI+fuzzy systems ([120]) combine domain knowledge with data-driven learning, improving interpretability and generalizability in data-scarce or high-risk applications.

### 6.2. Limitations and Gaps in Current Research

Despite promising progress, several specific and evidence-based limitations constrain real-world deployment and long-term scalability:

- Model Complexity vs. Edge Deployment Feasibility: High-accuracy models such as Transformers (e.g., DSFormer [101], TranDRL [158]) and hybrid CNN-LSTM architectures achieve superior predictive performance but require substantial computational resources, memory, and power—often exceeding the capabilities of typical edge hardware like Raspberry Pi, ESP32, or ARM Cortex-M4 microcontrollers used in many IIoT deployments [117,149]. This creates a practical gap where state-of-the-art AI models cannot be deployed in the resource-constrained environments they are intended to monitor.
- Lack of Standardized, Multi-Objective Evaluation: The reviewed literature reveals inconsistent and incomplete reporting of performance metrics. While prediction accuracy (e.g., F1-score, RMSE) is commonly reported, critical system-level indicators—such as end-to-end latency (including edge processing time), energy consumption per inference, model update overhead, and communication cost—are frequently omitted [166,170]. For instance, while [167] reports 92% accuracy and 72-h edge autonomy, the energy cost of continuous inference is not detailed, making it difficult to assess real-world viability for battery-powered sensors.
- Data Quality and Label Scarcity in Industrial Settings: Many high-performing supervised and deep learning models [36,153] assume the availability of large, clean, labeled datasets—a condition rarely met in real industrial environments where fault data is sparse, noisy, and expensive to annotate. Although unsupervised [40] and self-supervised [81] methods offer alternatives, they often trade interpretability and reliability for data efficiency, particularly in safety-critical applications where explainable predictions are mandatory.
- Security and Privacy Overheads in Distributed AIoT: While federated learning (FL) and edge-cloud collaboration enhance data privacy, they introduce significant, often underreported, overheads. FL frameworks like [172,176] must manage encrypted model updates, secure aggregation, and defense against poisoning attacks—processes that increase communication latency, computational load on edge nodes, and system complexity. Moreover, end-to-end security architectures that protect the entire AIoT stack from sensor to cloud remain conceptual rather than implemented in most studies [133].
- Limited Support for Dynamic Environments and Model Drift: Only a minority of reviewed approaches [174,175] explicitly address evolving operational conditions, sensor degradation, or unforeseen failure modes. Continuous learning, online model adaptation, and drift detection mechanisms are underexplored, especially in long-term deployments where equipment behavior and environmental factors change over time. This gap limits the longevity and autonomy of AIoT-PdM systems in dynamic industrial settings.
- Human Factors and Trust in Autonomous Recommendations: Most studies focus on algorithmic performance, with scant attention to human-AI interaction. Even when explainability tools like SHAP or LIME are incorporated [151], their effectiveness is rarely evaluated with actual maintenance operators in real workflow contexts. The trust calibration between human experts and AI-driven recommendations—and the design of intuitive, actionable interfaces—remains a critical but largely unaddressed challenge for field deployment [146].
- Integration Complexity and Brownfield Deployment Costs: Deploying AIoT in existing industrial infrastructure (brownfield) presents nontrivial challenges in sensor retrofit, power supply, network integration, and legacy protocol translation [127,143]. The cost and disruption of such integration are often prohibitive for small and medium enterprises, slowing adoption despite demonstrated technical benefits.

### 6.3. Future Perspectives and Research Opportunities

To address the limitations identified in Section 6.2 and to advance AIoT-PdM toward industrial readiness, the following concrete and actionable research directions are recommended:

- Develop Lightweight, Explainable Transformer Variants for Edge Deployment: Future work should focus on creating compressed, quantized, or distilled Transformer architectures (e.g., building on DSFormer [101] or STAR [103]) that maintain high RUL and FDD accuracy while operating within the memory and power constraints of microcontrollers such as ARM Cortex-M4 or Raspberry Pi [117,149]. These models should integrate intrinsic explainability mechanisms (e.g., attention heatmaps, saliency maps) validated through user studies with maintenance technicians to measure trust and decision accuracy. Application example: A real-time bearing fault diagnosis system deployed on an ESP32-based vibration monitor in a wind turbine, providing both fault alerts and visual explanations of which frequency bands contributed to the diagnosis.
- Establish a Standardized Multi-Objective Benchmark Suite for AIoT-PdM: The research community should collaborate to define an open benchmark that mandates reporting of both predictive performance (accuracy, F1, RMSE) and system-level metrics (end-to-end latency, energy per inference, communication overhead, model update cost). This benchmark should include diverse industrial datasets (e.g., C-MAPSS, NASA bearing, Tennessee Eastman Process) and reference edge hardware (Raspberry Pi, NVIDIA Jetson, ESP32) to enable reproducible and comparable evaluations [166,167]. Application example: A benchmark challenge for predictive maintenance of CNC machines, requiring participants to submit models that are evaluated not only on RUL prediction error but also on inference time per sample and energy consumption on a Raspberry Pi 4.
- Advance Semi-Supervised and Synthetic Data Methods for Industrial Data Scarcity: Research should prioritize semi-supervised learning frameworks and robust synthetic data generation (e.g., using diffusion models or physics-informed GANs) that are explicitly validated against distribution shift and data leakage. These methods must be evaluated not only on accuracy but also on their ability to generalize across machines, operating conditions, and fault types [40]. Application example: A semi-supervised anomaly detection system for rare pump failures in chemical plants, using a small set of labeled faults combined with a large corpus of unlabeled operational data, tested across multiple pump models and fluid types.
- Design Secure, Efficient Federated Learning Protocols for Heterogeneous IIoT Networks: New FL protocols should reduce communication overhead while providing verifiable defense against model poisoning and inference attacks. Techniques such as hybrid federated–split learning, adaptive client selection, and lightweight homomorphic encryption should be tested in real multi-site industrial environments [172,176]. Application example: A federated RUL prediction system for a fleet of electric buses across multiple cities, where each depot trains local models on its own data, and a global model is aggregated without exposing sensitive operational patterns or location data.
- Implement Continuous Learning and Drift Detection for Long-Term Autonomy: AIoT-PdM systems require online learning mechanisms that can adapt to sensor drift, new failure modes, and changing operating conditions without full retraining. Research should explore replay-based continual learning, Bayesian uncertainty estimation, and automated drift detection triggers that operate efficiently at the edge [174,175]. Application example: An edge-based motor health monitoring system that continuously updates its vibration analysis model as the motor ages and environmental conditions change, with automatic alerts when prediction confidence drops below a threshold indicating potential model drift.
- Conduct Human-in-the-Loop Studies for Trust and Adoption: Beyond technical metrics, research must evaluate how maintenance operators interact with AIoT recommendations. Controlled studies should measure the impact of explainable AI (XAI) interfaces (e.g., SHAP, LIME) on decision speed, error rates, and trust calibration in real or simulated maintenance scenarios [146,151]. Application example: A comparative study in a manufacturing plant where maintenance teams use two versions of a PdM dashboard—one with basic alerts and another with integrated SHAP-based explanations—measuring time-to-repair, false alarm response, and operator confidence surveys over a six-month period.
- Create Modular, Retrofit-Friendly AIoT Packages for Brownfield Integration: To lower adoption barriers, future work should develop modular hardware/software packages that simplify sensor retrofit, legacy protocol translation (e.g., MODBUS to MQTT), and incremental deployment in brownfield environments. Cost–benefit analyses and case studies in small and medium enterprises should accompany technical demonstrations [127,143]. Application example: A plug-and-play IIoT kit for legacy injection molding machines, including vibration sensors, a gateway that converts PLC data to MQTT, and a cloud dashboard that requires no programming from the maintenance team, demonstrated in a small automotive parts factory.

Overall, the convergence of AI and IIoT into AIoT systems presents a transformative opportunity for predictive maintenance. However, achieving robust, interpretable, and scalable implementations will require focused research on the specific technical, evaluative, and human-centered challenges outlined above. As Industry 5.0 continues to emphasize resilience, sustainability, and human-centricity, AIoT must evolve toward systems that are not only intelligent but also deployable, trustworthy, and integrally aligned with real-world industrial operations.

## 7. Conclusions

To ensure a coherent synthesis of our review, we revisit the research questions that guided this study. RQ1, which focused on recent advancements in AIoT-based predictive maintenance aligned with Industry 5.0, was addressed through a structured analysis of state-of-the-art techniques, including transformer models, federated learning, and Edge–Cloud collaboration. RQ2 explored the integration of artificial intelligence and IIoT technologies across industrial sectors; this was examined in depth through architectural comparisons, sectoral use cases, and a taxonomy of enabling technologies. RQ3, targeting challenges, limitations, and future perspectives, was discussed comprehensively, highlighting issues such as model interpretability, data quality, infrastructure complexity, and open research avenues in explainable, adaptive, and prescriptive AIoT frameworks.

The convergence of Artificial Intelligence and the Industrial Internet of Things—AIoT—has ushered in a new era for predictive maintenance, enabling data-driven decision-making, real-time monitoring, and intelligent fault detection across critical industrial domains.

This survey provided a structured overview of AIoT-based predictive maintenance, detailing its application across transportation, energy, manufacturing, and other industrial sectors. By analyzing diverse AI techniques, computing models, IoT architectures, and collaborative frameworks such as edge–cloud and federated learning, this study identified prevailing trends and system characteristics.

Key advantages of AIoT include improved prediction accuracy, scalability across heterogeneous environments, enhanced edge intelligence, and potential for real-time, human-in-the-loop optimization aligned with Industry 5.0 values. However, limitations such as interpretability gaps, model drift, integration complexity, and evolving security challenges remain significant.

Future work must address these limitations through lightweight, explainable, and continuously learning AI models; robust federated and prescriptive frameworks; and standardized benchmarks to ensure replicability. The continued evolution of AIoT is expected to deliver more resilient, sustainable, and intelligent maintenance ecosystems for the industries of tomorrow.

## Figures and Tables

**Table 1:** Mapping of Survey Contributions to Manuscript Structure.

Contribution	Section(s)	Primary Content/Thematic Focus
C1: Industry 5.0 and AIoT Foundations	Section 3	Overview of Industry 5.0 pillars, evolution of maintenance strategies, strategic role of AIoT in PdM.
C2: Unified AIoT Taxonomy and Background	Section 4	AI techniques (ML, DL, generative AI), IIoT components (sensors, communication, Edge–Cloud), AIoT integration architecture.
C3: AI & IIoT Techniques Review	Section 5.1 & Section 5.2	AI for Fault Detection and Diagnosis (FDD), RUL, scheduling; IIoT implementations in manufacturing, transportation, energy.
C4: AIoT-Enhanced Maintenance Paradigms	Section 5.3.1	Condition-Based Maintenance (CBM), Prognostics and Health Management (PHM), Prescriptive Maintenance, Hybrid AIoT strategies.
C5: Next-Gen AIoT Architectures	Section 5.3.2	Edge–Cloud Collaboration, Self-Adaptive & Federated Learning, Digital Twin-driven PdM.
C6: Synthesis and Future Directions	Section 6	Advantages, limitations, open challenges, and future research opportunities.

**Table 2:** Comparison of Communication Technologies for Predictive Maintenance.

Technology/Feature	Ethernet	Wi-Fi	LoRaWAN	5G
Communication Type	Wired	Wireless	Wireless	Wireless
Range	Local (LAN: typically up to 100 m)	Medium (indoor: 50–100 m; outdoor: up to 200 m)	Long-range (rural: up to 15 km; urban: 2–5 km)	Very long-range (with infrastructure: up to 100 km)
Data Transfer Rate	High (10 Mbps–10 Gbps)	Moderate (50 Mbps–1 Gbps)	Low (0.3–50 kbps)	Very High (100 Mbps–10 Gbps)
Latency	Low (typically 1–10 ms)	Moderate (typically 5–50 ms)	High (typically 1–3 s)	Ultra-low (typically 1–10 ms)
Power Consumption	High	Moderate	Very Low	Low
Bandwidth	High	Moderate	Low	Very High
Scalability	High	Moderate	High	High
Deployment Complexity	Medium (cabling required)	Low (easy to deploy)	High (needs specialized gateways)	High (requires new infrastructure)
Cost	Medium to High	Low to Medium	Low	High
Typical PdM Use Cases	Real-time monitoring of fixed machinery, high-bandwidth sensor data transfer	Factory/warehouse equipment monitoring, mobile data collection in industrial facilities	Remote asset monitoring (wind farms, pipelines, agriculture), low-power sensor networks	Mobile/autonomous equipment monitoring, real-time video analytics, remote diagnostics

Note: Values represent typical operational ranges under standard industrial conditions. Actual performance may vary based on environmental factors, network configuration, and implementation.

**Table 3:** Comparison of Communication Protocols and Standards for Predictive Maintenance.

Protocol/Feature	MQTT	OPC UA	Modbus	RESTful API
Type	Publish/Subscribe	Client/Server	Request/Response	Web-based API
Data Format	Lightweight messages (JSON, binary)	Structured industrial data model	Simple register-based	JSON/XML
Security	TLS encryption (v3.1+)	Strong security (encryption, authentication, certificates)	Weak (no built-in encryption; requires external security)	HTTPS/TLS, OAuth 2.0
Scalability	High (supports millions of devices)	High (enterprise-level scalability)	Low (limited to 247 devices per network)	High (stateless, horizontal scaling)
Latency	Low (typically 10–100 ms)	Low to Medium (typically 50–200 ms)	Low (typically 10–50 ms)	Medium (typically 100–500 ms)
Typical PdM Use Cases	Sensor-to-cloud communication, real-time monitoring, edge-to-cloud messaging	Industrial automation, machine data exchange, digital twins, cross-platform interoperability	Legacy industrial system integration, PLC monitoring, equipment monitoring in brownfield environments	Cloud-based dashboards, remote diagnostics, mobile access, web-based configuration interfaces

Note: Characteristics are based on typical industrial IoT implementations. Security and performance depend on specific configuration, network conditions, and versioning.

**Table 4:** Comparison of Edge Devices for Predictive Maintenance.

Attribute	Industrial Gateways	Edge Computers (Industrial PCs)	PLCs	Single-Board Computers	Edge AI Devices
Key Features	Data aggregation, protocol translation, cloud connectivity, network bridging	High-performance computing, advanced analytics, rugged design, multi-core processing	Control of machinery, data collection, real-time processing, deterministic operation	Low-cost, flexible, customizable, lightweight, open-source ecosystems	AI processing, machine learning acceleration, real-time decision making, neural network inference
Processing Capability	Basic to Moderate (ARM Cortex-A, 1–4 cores)	High (Intel Core i7/Xeon, 4–16 cores)	Moderate to High (real-time processors, 1–4 cores)	Low to Moderate (ARM Cortex-A, 1–4 cores)	High (AI-focused; GPU/TPU, 4–8 cores)
Connectivity	Ethernet, Wi-Fi, Cellular (4G/5G), Modbus, PROFINET	Ethernet (10 GbE), Wi-Fi 6, USB, PCIe	Ethernet, Modbus, PROFINET, CAN bus, RS-485	Ethernet, Wi-Fi, Bluetooth, GPIO, I^2^C, SPI	Ethernet, Wi-Fi 6, 5G, PCIe, USB 3.0
Cost Range	Medium to High ($200–$1000+)	High ($1000–$5000+)	Medium ($500–$2000)	Low to Medium ($50–$200)	Medium to High ($300–$2000+)
Examples	Moxa UC-8100, Siemens IoT2040, HPE Edgeline	Advantech IPC, Beckhoff C6015, Dell Edge Gateway	Siemens S7-1500, Allen-Bradley Logix, Schneider Electric Modicon	Raspberry Pi 4, BeagleBone Black, Arduino Portenta	NVIDIA Jetson, Google Coral Edge TPU, Intel Movidius
Typical PdM Use Cases	Aggregating sensor data from multiple machines, protocol conversion, secure cloud uplink	Real-time analytics, local anomaly detection, predictive modeling, video analytics for quality inspection	Monitoring PLC-controlled equipment, vibration analysis, temperature/pressure monitoring	Prototyping PdM solutions, small-scale monitoring, educational/research deployments	Real-time fault classification, predictive maintenance at the edge, AI-driven anomaly detection

Note: Specifications represent typical commercial models available as of 2025. Performance, connectivity, and cost may vary based on manufacturer, configuration, and regional availability.

**Table 5:** Overview of IIoT Frameworks for Predictive Maintenance: Descriptions, Applications, Strengths, and Challenges.

Framework	Description	Applications in PdM	Strengths	Challenges
MTConnect	Open-source standard for manufacturing, facilitating data communication between devices.	Enables seamless integration of machine data for PdM analytics.	Standardized data model, vendor-neutral, real-time data access.	Limited adoption outside manufacturing; requires additional tools for analysis.
Vanguard Predictive Maintenance Framework	Proprietary framework integrating IoT devices and analytics for PdM.	Optimized for PdM; offers predictive insights using ML algorithms.	Focused on PdM, scalable, offers advanced analytics.	Proprietary, higher cost, dependent on specific tools.
Edge Computing Frameworks	Decentralized computation model processing data close to devices.	Reduces latency in PdM by processing data at the edge for real-time analysis.	Low latency, efficient bandwidth use, better for real-time scenarios.	Complexity in deployment and maintenance, high upfront cost.
OpenIoT	Open-source middleware platform connecting IoT devices and applications.	Facilitates data collection and analysis for PdM through interoperability.	Open-source, customizable, supports various IoT devices.	Requires technical expertise for deployment and customization.
M2M Communication Frameworks	Technologies enabling direct communication between machines.	Supports real-time data exchange and monitoring for PdM.	Highly efficient in communication, enables real-time diagnostics.	Limited to communication, not comprehensive for analytics.
Node-RED	Flow-based development tool for IoT, connecting devices and applications through visual workflows.	Simplifies the creation of PdM workflows, integrating sensors and analytics.	Easy to use, supports many plugins, ideal for rapid prototyping.	Limited scalability, performance issues in large-scale deployments.
IoTivity	Open-source IoT framework for ensuring device-to-device communication.	Enables interoperability between devices for data collection and PdM.	Standardized, secure, facilitates seamless communication between IoT devices.	Requires adaptation for specific PdM applications, less focus on analytics capabilities.

**Table 6:** Summary of Supervised Learning Approaches for Fault Detection.

Study	Techniques Used	Key Contributions	Performance Metrics	Limitations
[33]	Logistic Regression, LDA, QDA, SVC, Random Forest, Gradient Boosting, ANN	SVC outperformed others in precision-recall metrics.	High precision-recall for SVC.	LDA and QDA showed lower accuracy.
[34]	Decision Trees, Random Forests, SVM, k-NN, ANN	Random Forests and ANN showed high accuracy.	High accuracy for Random Forests and ANN.	SVM required high computational resources.
[35]	Random Forests, Decision Trees, k-NN	Random Forests excelled on smaller datasets, k-NN on larger datasets.	Random Forests and k-NN performance varied by dataset size.	Performance depended on dataset size and structure.
[36]	Random Forest, GBM, DNNs	DNNs demonstrated superior performance in industrial settings.	DNNs outperformed others in identifying key failure predictors.	DNNs required extensive training data and tuning.
[37]	Decision Tree, Gaussian Naive Bayes, Gaussian Process Classifier, SVM	GPC achieved the highest performance.	GPC: 99.56% accuracy, 0.978 precision, 0.989 recall, 0.983 F1 score, 0.99 AUC.	GPC is computationally expensive.

**Table 7:** Summary of Unsupervised Learning Approaches for Fault Detection.

Study	Techniques Used	Key Contributions	Performance Metrics	Limitations
[38]	One-Class SVM, Isolation Forest, Local Outlier Factor	LOF demonstrated the best trade-off in sensitivity and specificity.	LOF: 77.6% sensitivity, 72.1% specificity, 0.81 ms inference time.	Lower specificity compared to supervised methods.
[39]	PCA T^2^ statistic, hierarchical clustering, K-means, fuzzy C-means, model-based clustering	PCA T^2^ statistic and model-based clustering were most effective.	PCA T^2^ and model-based clustering identified faults effectively.	Clustering techniques require careful parameter tuning.
[40]	Profile-Based (PB) anomaly detection	PB method evaluated deviations from operational profiles.	Anomaly scores influenced by material changes and maintenance activities.	Sensitivity to operational variations may lead to false positives.
[41]	Kernel Spectral Clustering (KSC)	KSC handled high-dimensional sensor data effectively.	KSC outperformed traditional clustering techniques.	High computational cost for large datasets.
[42]	Dimensionality reduction within CRISP-DM framework	Improved anomaly detection and diagnostic analysis.	Enhanced maintenance decision-making.	Dimensionality reduction may lead to loss of information.
[43]	Data augmentation with soft contrastive learning	Increased sensitivity to subtle anomalies.	Average performance score of 57.5, enhanced version (USD*) reached 64.4.	Performance highly dependent on dataset characteristics.
[44]	OC-SVM, Minimum Covariance Determinant, Majority Voting Ensemble	Ensemble approach yielded the highest accuracy.	Ensemble approach achieved the highest accuracy.	Requires multiple models, increasing complexity and computation.

**Table 8:** Summary of Reinforcement Learning Approaches for Fault Detection.

Study	Techniques Used	Key Contributions	Performance Metrics	Limitations
[45]	Constrained Reinforcement Learning (CRL)	FIERL improved fault detection efficiency and robustness.	Faster and more accurate fault diagnosis compared to traditional methods.	Requires complex constraint handling and extensive tuning.
[46]	Deep Reinforcement Learning (DRL)	Achieved over 99% diagnosis accuracy with small datasets.	Outperformed SVM, CNN, and GRU.	Limited generalization due to small dataset size.
[47]	Deep Q-Network (DQN)	High accuracy in identifying fault types and health states.	Outperformed traditional methods under variable operating conditions.	Requires significant computational resources for training.
[48]	RL-based state-space model	Expanded tolerable fault range and improved system performance.	Superior to traditional fault-tolerant control methods.	High complexity in state-space modeling and control law derivation.

**Table 9:** Summary of Deep Learning Approaches for Fault Detection.

Study	Techniques Used	Key Contributions	Performance Metrics	Limitations
[49]	LSTM, CNN-based autoencoders	Achieved low inference times and minimal memory requirements.	Suitable for real-time applications.	May struggle with highly complex acoustic environments.
[50]	Causal Disentanglement Hidden Markov Model (CDHM)	Achieved 100% accuracy in workload transfer scenarios.	Outperformed DDC and DANN.	Requires carefully designed disentanglement strategies.
[51]	Hybrid CNN-RNN framework	Higher accuracy and faster processing times.	Outperformed existing methods.	Computationally intensive due to hybrid architecture.
[52]	Autoencoder neural networks, CNNs	High performance in anomaly detection and classification.	Achieved high performance in fault diagnosis.	Performance highly dependent on data quality and preprocessing.
[53]	CNNs, LSTMs	Captured spatial and temporal patterns in sensor data.	F-Scores of 92% and 97% for binary and multiple classification tasks.	Requires large datasets for optimal performance.
[54]	Hybrid DCNN-SVM approach	Improved diagnostic accuracy to 98.71%.	Integrated expert knowledge for better accuracy.	Model interpretability remains a challenge.
[55]	CNNs for thermal imaging	CNNs demonstrated superiority over ANNs in feature extraction.	Effective non-invasive bearing fault diagnosis.	CNNs require extensive labeled data for training.
[56]	1D-CNN optimized via reinforcement-learning-based NAS	Superior accuracy and interpretability.	Achieved superior accuracy in fault detection.	NAS optimization is computationally expensive.
[57]	Advanced CNN-based model	Achieved over 99.9% accuracy with optimized computational efficiency.	High accuracy and computational efficiency.	High accuracy may not generalize well to unseen fault types.

**Table 10:** Summary of Generative AI Approaches for Fault Detection.

Study	Techniques Used	Key Contributions	Performance Metrics	Limitations
[58]	Generative Adversarial Networks (GANs)	Improved model performance with synthetic fault signals.	99.41% accuracy with 20% real data, 93.1% with synthetic data.	Requires careful tuning of generator-discriminator balance.
[59]	GAN-based framework	Achieved 100% accuracy on CWRU and laboratory datasets.	High accuracy on imbalanced time series data.	Computationally expensive training process.
[60]	Generative Adversarial Wavelet Neural Operator (GAWNO)	High accuracy in fault detection for multivariate time series.	Demonstrated high accuracy in fault detection.	Limited scalability to different fault types.
[61]	Graph Wavelet Autoencoder (GWAE), Graph Wavelet Variational Autoencoder (GWVAE)	Achieved 3–4% performance improvement.	Improved multiscale feature extraction.	Performance dependent on graph structure selection.
[62]	Variational Autoencoders (VAEs)	Achieved 98.5% accuracy on CWRU dataset.	Combined MSE and KL divergence for better performance.	Requires extensive hyperparameter tuning.
[64]	BiLSTM-VAE model	Achieved 98% accuracy on SKAB and TEP datasets.	Outperformed traditional methods in handling imbalanced data.	Susceptible to overfitting on small datasets.
[66]	VAE-LSTM hybrid model	Achieved 95% fault detection rate on Tennessee Eastman Process dataset.	Outperformed PCA and standalone LSTM.	High computational cost due to hybrid architecture.
[62]	PSO-ConvLSTM-Transformer model	Achieved 98.75% accuracy in wind turbine blade icing fault detection.	High accuracy through feature engineering and hyperparameter optimization.	Complex model architecture requiring significant tuning.
[67,68]	Transformer-based models	Achieved 98.5% and 99.5% accuracy on benchmark datasets.	Robust and interpretable fault diagnosis.	Requires large labeled datasets for optimal performance.
[69]	Transformer Neural Network (TNN)	Achieved 96.2% accuracy in power transformer fault diagnosis.	Rapid fault response times.	High memory consumption.
[72]	Transformer with inter-variable attention	Achieved state-of-the-art performance on benchmark datasets.	High accuracy in anomaly detection for multivariate time series.	Requires careful feature selection and preprocessing.
[73]	LSTM-Autoencoders, Transformer Encoders	Achieved F1-score of 0.92 in anomaly detection.	High precision in failure prediction.	Sensitivity to hyperparameter selection.

**Table 11:** Summary of Machine Learning Approaches for RUL Prediction.

Study	Techniques Used	Key Contributions	Performance Metrics	Limitations
[74]	STFT, Envelope Analysis, SVM, k-NN	Vibration signal analysis for feature extraction, wear-state classification.	74.3% accuracy, MAE = 0.08.	Data preprocessing complexity.
[75]	Six ML models (including LGBM)	LGBM outperformed others on aviation datasets.	AUC = 89%.	Model selection sensitivity.
[76]	SVR, LSTM, Grey Wolf Optimization	Hybrid model reducing RUL over-estimation.	Improved real-time applicability.	High computational cost.
[77]	RF, XGB, MLP, SVR	RF prevented 42% of production line failures.	RF superior in fault prevention.	Limited dataset variability.
[78]	Regression, ANN	Vibration signal features capture degradation trends.	Effective for wind turbines, test rigs.	ANN interpretability issues.
[79]	GBT, RF	GBT: 93.91% accuracy, RF: 91.78%, RF efficient.	High prediction accuracy, efficiency.	Potential overfitting in GBT.
[80]	Bayesian Filtering	Improved aircraft engine prognostics.	RMSE improved by 34.5–55.6%.	Sensitivity to prior assumptions.
[81]	Self-Supervised Learning	Pre-training on unlabeled data.	RMSE reduced by 10–15%.	Generalization across conditions.
[82]	UKF, Adaptive Kalman Filter	Dynamic noise adjustment in RUL prediction.	Lower estimation errors, fluctuations.	Complexity in noise modeling.

**Table 12:** Summary of Deep Learning Approaches for RUL Estimation.

Study	Techniques Used	Key Contributions	Performance Metrics	Limitations
[83]	CA-DANN	Mitigates domain shifts in variable speed conditions using synthetic data	Improved RUL accuracy under speed variations	Requires synthetic data generation, complexity in implementation
[84]	CNN-LSTM	Feature extraction from three-phase voltage and current signals for AC contactors	RMSE = 54.7, MAE = 51.8 (outperforms SVM, CNN, LSTM)	Higher complexity compared to standalone models
[85]	CNN + HHT + ε-SVR	Extracts nonlinear degradation indicators for bearings	High accuracy in degradation trend estimation	Computationally intensive
[86]	SCBNet	Integrates Adjacent Backbone Assembly Strategy for improved RUL estimation	Enhanced feature extraction with low computational cost	Requires careful hyperparameter tuning
[87]	LSTM	Predicts UAS propulsion failures using "mean peak frequency” indicator	RMSE = 3.7142 Hz (4 s), 1.4831 Hz (10 s), 1.3455 Hz (10 s)	Limited to specific degradation indicators
[88]	MSWR-LRCN	Multi-scale feature fusion and attention-based residual shrinkage for noise reduction	Superior noise handling and prediction accuracy	Increased architectural complexity
[89]	SSAE + Logistic Regression	Feature extraction and fusion from multi-sensor data	Optimized performance using grid search	Requires extensive parameter tuning
[90]	DNN + Denoising Autoencoder	Two-stage model: health classification + RUL estimation	Improved robustness to sensor noise	Complexity increases with added stages
[91]	MLP + PCA + Interpolation	Dimensionality reduction and missing data handling for RUL estimation	Training RUL MSE: 21-94, Validation RUL MSE: 509-1427	Performance varies across datasets
[92]	BiLSTM + Feature Selection	Two-stage feature selection with change point detection	27.8% accuracy improvement on C-MAPSS dataset	Requires extensive preprocessing
[93]	TDDN (1D CNN + Attention)	Extracts temporal degradation patterns for machinery	Improved generalization to unseen failure modes	High training data requirement
[94]	Bayesian MLP + RF + SES	Optimized feature selection and smoothing for RUL	6.1% RMSE reduction on FD001 test set	Requires Bayesian optimization overhead

**Table 13:** Summary of Generative AI Approaches for Predictive Maintenance.

Study	Techniques Used	Key Contributions	Performance Metrics	Limitations
[95]	Recurrent Variational Autoencoder (RVAE)	Captures temporal dependencies and uncertainties in high-dimensional sensor data.	Outperforms traditional ML and DL models.	Computationally intensive.
[96]	Variational Autoencoder (VAE) with regression	Uses VAE for feature extraction followed by a regression model for RUL estimation.	Handles noisy industrial data effectively.	Sensitive to hyperparameter tuning.
[97]	CMG-VAE (TCN + Graph Representation Learning)	Models structural relationships in spacecraft telemetry data.	24% reduction in RMSE over baselines.	Requires extensive dataset preprocessing.
[98]	VAE + GAN + LSTM	Improves degradation trend learning without predefined failure thresholds.	Enhances feature selection and degradation tracking.	Complex model training and optimization.
[99]	DTC-VAE + MMA-LSTM	Incorporates degradation-trend constraints for reliable RUL prediction.	Effective for rotary machinery.	Computationally expensive.
[100]	Transformer-based Encoder-Transformer model	Inspired by LLMs, achieving a significant performance improvement.	137.65% improvement over previous approaches.	High computational demands.
[101]	DSFormer (Dual-Attention + TCN + Feature Decomposition)	Improves RMSE and Score metrics for predictive maintenance.	3.2% improvement in RMSE, 2.5% in Score.	Requires substantial training data.
[102]	DAST (Dual Aspect Self-Attention Transformer)	Enhances cross-sensor and temporal feature extraction.	Outperforms state-of-the-art models in time-series data.	High memory requirements.
[103]	STAR (Two-stage Attention)	Addresses temporal and sensor-wise dependencies.	RMSE: 10.61 (dataset1), 13.47 (dataset2), 10.71 (dataset3), 15.87 (dataset4).	Model complexity may hinder real-time applications.
[104]	SAConvFormer (CNN + Transformer)	Analyzes raw vibration data for superior RUL prediction.	Enhanced RMSE and MAE accuracy.	Requires large-scale computational resources.
[105]	LECformer (LECSA-enhanced Transformer)	Dynamically weights sensor channels for long-term dependencies.	Improved spatial correlation modeling.	Challenging to fine-tune for diverse datasets.

**Table 14:** Summary of Reinforcement Learning Approaches for Predictive Analytics and Maintenance Scheduling.

Study	Techniques Used	Key Contributions	Performance Metrics	Limitations
[106]	RL, Approximate Dynamic Programming	Optimized maintenance decisions in multistage production systems.	Cost reduction: 9.68% (SBP), 39.07% (TBP), 39.56% (GP); Throughput ↑ 9%.	High computational complexity.
[107]	Multi-Agent RL	Dynamic coordination of maintenance scheduling.	Machine downtime ↓ 75%.	Partial observability challenges.
[108]	Multi-Agent DRL, POMDP, Bayesian Inference	Risk-aware maintenance with historical dependencies.	Outperforms baselines in risk management.	Long-term resource constraints.
[109]	Hierarchical RL, MDPs	Coordination of maintenance in multicomponent systems.	Outperforms deep RL in gas plant and series systems.	Complexity in agent hierarchy.
[110]	Deep Q-Network (DQN), Gamma Process	Learning-based optimal policy for maintenance.	Cost reduction, improved reliability.	Sensitive to reward function design.
[111]	DRL for Rail Maintenance	Optimizing rail infrastructure maintenance and renewal.	Long-term cost savings.	Uncertainty in safety constraints.
[112]	HMM + Deep RL	Two-level PdM for turbofan engines.	Better interpretability, outperforms standalone RL.	Complexity in integrating models.
[113]	Offline DRL, BCQ, CQL	Optimizing maintenance without real-time interaction.	Cost reduction, reliability improvements.	Handling noisy data in offline learning.

**Table 15:** Comparison of IIoT-Based Predictive Maintenance Systems in Smart Manufacturing.

Study	Sensors Used	Communication Technology	Processing Unit	Computing Model	Use Case
[114]	Temp., Vibration, Acoustic, Pressure	Wi-Fi, Zigbee	Raspberry Pi + Cloud (AWS/Google)	Edge + Cloud AI	Industrial Machinery
[115]	Vibration, Temperature, Current	Wi-Fi Mesh, MODBUS RTU	ESP32-WROOM-32UE	Edge + Cloud Dashboard	Industrial Machines
[116]	Vibration, Current, Torque	MQTT	Central Platform + Cloud	SPC + Deep Learning	Electric Motors, Transfer Vehicles
[117]	Vibration, Temp., Sound, Current/Voltage	BLE, LoRaWAN, Wi-Fi	ARM Cortex-M4	Embedded Edge AI	General Manufacturing
[118]	Vibration, Current, Temperature	MQTT	IoT Devices + Azure Cloud	Cloud DSS + ML	Industrial Maintenance Optimization
[119]	Load Cells, Pressure, Power/Voltage	MQTT	IoT Edge DAQ Device	On-Prem Server + ML	Laser Plastic Welding
[120]	Temp., Current, Pressure, Vibration, Gap	MQTT	Central IoT Gateway + Cloud	Cloud Predictive Analytics	Cardboard Production
[121]	Pressure	SPI Bus	Raspberry Pi 4 Model B	Local CSV Logging + Analysis	CNC TCM
[122]	Acoustic Emission, Vibration, Current	UMK-SE Cable	MIO-16 DAQ Board	RMS Signal Processing	CNC Prognostics
[123]	Vibration	Wired Ethernet	Raspberry Pi 3 Model B+	Centralized DB	CNC Real-Time Monitoring

**Table 16:** Comparison of IIoT-Based Predictive Maintenance Systems in Transportation.

Study	Sensors Used	Communication Technology	Processing Unit	Computing Model	Industry Use Case
[124]	Coolant Temp, Fuel Trim, MAF, Throttle Position	NB-IoT, MQTT	Raspberry Pi Zero WH	Cloud (ThingsBoard)	Vehicle Diagnostics
[125]	Speed, Vibration, Engine Load	5G	OBD-II + ECU	Cloud + External Data Integration	Automotive Telematics
[126]	Temperature, Pressure	Corporate Ethernet	Smart Observer System	Real-Time Monitoring	Automotive Manufacturing
[127]	Temperature, Vibration, Current	Wi-Fi 5GHz	Banana Pi	Azure IoT + ML Models	BMW Heavy-Lift Monorails
[128]	Brake Pressure	Wireless (unspecified)	ECU + ThingWorx Platform	Digital Twin (CREO Simulate)	Automotive Brake Pads
[129]	Engine Pressure, Temperature	ECU Logging + Remote Storage	ECU + Cloud Platform	Real-Time Monitoring	Turbocharged Engines
[130]	Temp, Battery Voltage, Brake Wear	Wireless IoT (unspecified)	Cloud Analytics Platform	ML + Cloud Dashboards	Sustainable Transport Fleets
[131]	Engine Temp, Battery Voltage, Tire Pressure, Brake Pads, Oil Level	Wireless IoT (unspecified)	Cloud-Based Platform	Dynamic Maintenance Scheduling	Public/Commercial Transport
[132]	Fuel, Coolant, Oil Status, Mileage	MQTT	Eclipse Kura Gateway	Cloud (Kafka, Storm, Hadoop)	Connected Cars
[133]	Temp, Fuel, Vibration, GPS, Speed	Wireless + 6G + Blockchain	IoT-ILTMF System	AI + Cloud + Digital Twin	Vehicular Logistics Systems
[134]	Temp., Vibration, Energy Consumption	CAN-FD, Mesh, 802.11p, LTE-M/NB-IoT	Edge Computers + Fog + Cloud	Edge–Fog–Cloud Architecture	Green Transit Systems (EVs, Hydrogen)
[135]	Hydraulic Pressure	Wireless (unspecified)	Edge Gateways + Cloud	Edge–Cloud Collaboration	Smart Port Container Handling

**Table 17:** Comparison of IIoT-Based Predictive Maintenance Systems in Energy Sector.

Study	Sensors Used	Communication Technology	Processing Unit	Computing Model	Industry Use Case
[136]	Temperature, Vibration	6LoWPAN, RPL, CoAP	Multi-interface Gateway	Centralized (RCSR Analysis Room)	Power Plant Machinery Monitoring
[137]	Wind Speed, Direction, Temperature, Humidity	Wireless IoT (unspecified)	Microcontrollers	Cloud + ML Algorithms	Wind Turbine Optimization
[138]	Temperature, Vibration, Rotational Speed	Wi-Fi, GSM	Microcontroller Units	Cloud-Based Monitoring	Wind Turbine Predictive Maintenance
[139]	Gearbox Temp, Wind Speed, Ambient Temp, Generator/Rotator RPM	SCADA Integrated IoT	Centralized Analytics Platform	Predictive Models (Anomaly Detection)	Wind Turbine Maintenance
[140]	Differential Pressure, Acoustic	Edge Devices, LAN, MQTT	Embedded Network Connectors + PLCs	Cloud Dashboards	Oil and Gas Refinery Monitoring
[141]	Temp, Pressure, Power, Vibration	SCADA-Integrated IIoT	Cloud Platform (Digital Twin)	Cloud + Digital Twin Modeling	Wind Turbine Gearbox Monitoring

**Table 18:** Comparison of AIoT-Based Condition-Based Maintenance Approaches.

Article	Domain	AI Techniques	AIoT Architecture	Limitations
[142]	Smart Manufacturing	ML-based anomaly detection	MQTT + Edge + Cloud DSS	Edge devices have limited compute; requires reliable network for cloud sync
[143]	Aircraft Monitoring	Edge ML	Satellite sensors + 6G + Three-tiered AIoT	High complexity and cost; limited scalability in low-cost contexts
[144]	Automotive Systems	AI with edge service migration	5G-enabled edge computing	Strong reliance on 5G; limited testing in diverse mobility conditions
[145]	Wind Turbines	RUL prediction models	IoT + AI	Limited generalization to unseen failure modes; needs retraining
[146]	LNG Infrastructure	Conversational AI agents	Human-in-the-loop + IIoT	Latency from human-in-the-loop; needs operator training and trust
[147]	Firefighting Pumps	CNN-GRU deep learning	Real-time IoT monitoring	High dependency on data quality; limited generalization to other systems
[148]	ESP Systems	Decision Trees, Neural Nets, MOORA	AI-enhanced model selection	Real-time adaptation unclear; potential overfitting with static models
[149]	Wood-Residue Systems	ELM (Extreme Learning Machines)	IoT-based monitoring	Low interpretability; may need tuning for different conditions

**Table 19:** Comparison of AIoT-Based PHM Approaches.

Study	AI Techniques	IoT/Edge Components	Key Contributions/Outcomes	Limitations
[150]	Health state classification, RUL prediction	Multisensor IoT	Integrates reliability-centered maintenance with AI for adaptive decision-making	Limited to predefined fault categories; lacks dynamic model retraining on evolving data
[151]	Semi-supervised CNN, XGBoost, SHAP	Sensor network	Robust early fault detection with interpretable models	High dependency on labeled and quality sensor data; SHAP may not scale well with real-time streaming
[152]	Vibration-based AI analysis	IoT (triaxial accelerometers), Web/mobile interface	Full-stack AIoT SHM system with real-time visualization	Limited generalizability to different structures and materials; edge analytics not emphasized
[153]	PSO-CNN, GRU with Attention	IoT + Augmented Reality	Achieved ∼98.16% accuracy; reduced downtime via AR-assisted PHM	Computationally intensive; AR integration may not be cost-effective for small-scale factories
[154]	Foundation Models, Federated Learning	Edge AI devices, IoT	Ensures privacy and scalability through federated PHM learning	High infrastructure and synchronization requirements; foundation models may lack domain specificity
[155]	Rule-based fault logic	IoT sensors, GSM, Cloud	Low-cost deployment reducing downtime in real-world setups	Rule-based logic lacks adaptability; limited scalability for complex fault scenarios

**Table 20:** Comparison of AIoT-Driven Prescriptive Maintenance Studies.

Study	AI Techniques	IoT Role	Prescriptive Capabilities	Limitation
[156]	BiESN, BiLSTM	Real-time data acquisition and monitoring via embedded IoT	Fault diagnosis, RUL estimation, maintenance guidance	High model complexity may limit deployment on ultra-low-power or memory-constrained devices
[157]	Lightweight ML, Local Anomaly Detection	On-device edge analytics for autonomous decisions	Autonomous local decision-making without cloud reliance	Limited model sophistication may reduce accuracy due to edge hardware constraints
[158]	Transformer, DRL, RLHF	Federated sensing and model training across distributed IoT nodes	RUL prediction, policy optimization via DRL	High training complexity and communication overhead in federated environments
[159]	Deep Reinforcement Learning (DRL)	Centralized sensor data aggregation and learning loop integration	Adaptive and automated maintenance decisions	Centralized design may affect scalability and latency in large-scale deployments
[160]	Multi-source Data Fusion	Heterogeneous sensor integration for informed decision-making	Cost-effective, sustainable maintenance recommendations	Relies on static rules and lacks adaptive learning in dynamic environments

**Table 21:** Comparison of Hybrid AIoT-Based Maintenance Strategies.

Study	Hybrid Approach	AI Technique	IoT Integration	Key Features	Limitation
[118]	Multi-level data acquisition + ML layer	Random Forest	IoT-enabled process quality monitoring	Cloud-based incremental learning; no run-to-failure data needed	Limited explainability of ML outputs; indirect indicators may reduce generalizability
[161]	Physics-informed RNN with fleet data	PI-RNN	Fleet-level IoT operational data	Effective with partially observed damage; physics + data fusion	Depends on accuracy of physics assumptions (e.g., Paris’ law); less suited for non-fatigue failures
[162]	Hybrid PINN for lubricant/fatigue	Recurrent Neural Network	SCADA-like sensor integration	Optimizes maintenance using grease and fatigue models	Application-specific model design; requires expert calibration
[120]	Two-stage fuzzy logic + ANN	ANN + Fuzzy Logic	IoT sensor + operator data	Combines interpretability and learning; expert-informed decisions	Fuzzy rule base is subjective; limited scalability
[163]	Progressive hybrid (physics to data-driven)	Incrementally integrated ML	IoT sensors on cutting tools	Adaptive lifecycle modeling; starts with physics, evolves with data	Initial accuracy limited by physics model; requires data growth for full performance

**Table 22:** Comparison of Edge–Cloud Collaborative Intelligence Approaches for Predictive Maintenance in AIoT Systems.

Study	Architecture	AI Techniques	IoT Integration	Key Results	Limitations
[164]	Layered Edge–Cloud with DRL	Deep Reinforcement Learning (Policy at Edge, Training in Cloud)	Industrial IoT with distributed sensor nodes and real-time DRL-based control	Reduced latency, lower operational cost, improved uptime and anomaly detection	DRL training complexity; limited discussion on handling edge device failures
[165]	Task Scheduling among IoT Sensors, Edge, and Cloud	Deep Q-Network (DQN)	Smart sensors coordinated via energy-aware scheduling algorithms	Real-time fault prediction, bandwidth-efficient, energy-saving	Scalability to more complex IoT deployments not assessed; limited security evaluation
[166]	KNN at Edge, LSTM at Cloud	Lightweight KNN + LSTM	Industrial IoT with local anomaly detection and cloud-based trend analysis	35% latency and 28% energy reduction; 60% bandwidth saving	Reduced detection accuracy at edge; limited model adaptability to unseen fault types
[167]	Edge–Cloud PdM with Autonomous Edge Units	RUL Estimation + Anomaly Detection	IIoT system handling 850 GB/day from sensors for real-time processing	92% prediction accuracy, 23% OEE increase, 72 h autonomy, 31% cloud cost cut	High edge processing/storage requirements; update mechanism not detailed
[168]	Federated Edge–Cloud with OTA Updates	Federated Learning + Robust Gradient Descent	Heterogeneous IoT environment with local training at edge nodes	Low-latency, spectrum-efficient RUL/anomaly learning across diverse networks	FL sensitive to adversarial updates; communication overhead; OTA requires stable links
[169]	Hybrid Edge–Cloud MES (Pharma)	Local Anomaly Detection + Cloud Optimization	IIoT integrated in Manufacturing Execution System (MES)	50% fewer unplanned downtimes, 75% fewer breakdowns	Domain-specific implementation; limited detail on Edge–Cloud orchestration
[170]	QoS-Aware Edge Server Placement + FL	Federated Learning + Edge-based RUL	Smart IIoT network with server-placement strategy for latency-aware data routing	60% energy saving, 35% latency reduction, high prediction accuracy	Assumes optimal server placement; lacks fault tolerance for edge failures
[171]	IntelliPdM: Edge-Cloud Co-Inference	Edge ML + Cloud DL + Synthetic Data Fusion	Real-time IIoT sensors with ultra-low latency detection and global cloud analytics	95–99% accuracy, 10× ROI, cost and breakdown reduction	Synthetic data quality impact; privacy concerns in cloud-centric data fusion

**Table 23:** Comparison of AIoT-powered self-adaptive and federated learning approaches for Predictive Maintenance.

Study	AI Techniques	FL Features	IoT Integration	Performance	Limitation
[172]	ML/DL on time-series sensor data	Iterative global updates adapting to edge machine behavior	IIoT machines (temperature, vibration sensors)	97.2% accuracy, 40% reduction in communication overhead	No explainability; assumes reliable aggregation server
[173]	CNN-BiLSTM, ANN, RF, SVM	Global model updated from decentralized real-time data streams	IIoT across multiple manufacturing sites	98.15% accuracy, better than hybrid ML/FL baselines	Complex model orchestration; increased local processing load
[154]	Lightweight AI models fine-tuned from FMs	Adaptive local training (batch size, learning rate) based on resources	Aircraft edge devices (vibration, pressure, temperature)	Better accuracy, lower false alarms, faster convergence	Foundation model integration increases memory footprint
[174]	Lightweight autoencoder	Local models learn machine-specific behavior while contributing globally	IIoT for rotating machinery (vibration sensors)	Similar to centralized performance; reduced network usage	No global context modeling; limited anomaly interpretability
[175]	Knowledge distillation models	Dynamic loss balancing between distillation and hard labels	Distributed AIoT edge nodes	Outperforms FedAvg in accuracy and communication efficiency	Tuning distillation parameters is non-trivial
[176]	LSTM with SHAP-based temporal attention	Client weight adaptation via model divergence (L2 norm, cosine similarity)	IIoT in Industry 5.0 with 6G slicing	F1 = 0.93, <12 ms latency, robust to attacks	Complex implementation; high dependency on network quality

**Table 24:** Comparison of AIoT-Driven Digital Twin Frameworks for Predictive Maintenance.

Study	AI Techniques	IoT & Architecture	Digital Twin Functionality	Key Contributions	Limitations
[177]	Random Forest, XGBoost, SVR	IIoT sensors; cloud-based	Simulation and prediction of surface roughness and power	Two-cycle model selection and integration for CNC optimization	Specific to CNC turning; lacks real-time industrial deployment
[178]	Autoencoder + LSTM	Profinet IIoT; edge-connected	Anomaly detection from unlabeled time-series data	Real-time unsupervised predictive diagnostics	Sensitivity to noisy or dynamic operating conditions
[179]	ML models (unspecified)	Bi-directional IIoT; Edge–Cloud	Fault prediction, simulation, lifecycle forecasting	Intelligent maintenance of power systems with DT-AI integration	Lack of details on AI model implementation and validation
[180]	XGBoost, LSTM, Decision Tree	Fog + edge + cloud (ISO 23247)	Distributed analytics, anomaly detection, feedback control	Scalable DT with reduced latency and high accuracy for turbines	Integration complexity; high infrastructure cost
[181]	Random Forest, Decision Tree	Azure cloud DT; sensor + test data	Inspection, fatigue failure prediction	100% accurate predictive inspection using fused features	Limited to 3D-printed parts; lacks generalizability
[182]	Ensemble Kalman Filter, ML classifiers	IIoT + cloud; hybrid physics-AI	Fluid and structural modeling with real-time updates	Physics-informed AIoT-DT for smart pipelines	Domain-specific model tuning; high computational load
[183]	ContValueNet (NN)	Edge–server adaptive DTs	Offloading simulation and inference optimization	Dual-DT system for adaptive DNN in AIoT inference	Focuses on inference rather than failure detection
[184]	AI-based RUL estimation, fault classifiers	Wireless IIoT + Simulink + dSPACE	Fault simulation, real-time diagnostics	Lightweight DT for SCIM with accurate fault estimation	Hardware-specific; limited to electric motors

## Data Availability

The datasets analyzed in this study are publicly available.

## Supplementary Materials

The following supporting information can be downloaded at: https://www.mdpi.com/article/10.3390/s25247636/s1, Table S1: PRISMA 2020 Checklist used in the current systematic review study.

## Abbreviations

AFD	Active Fault Detection
AI	Artificial Intelligence
AIoT	Artificial Intelligence of Things
ANN	Artificial Neural Network
ATAM	Architecture Tradeoff Analysis Method
BERT	Bidirectional Encoder Representations from Transformers
CA-DANN	Context-Aware Domain Adversarial Neural Network
CBM	Condition-Based Maintenance
CDHM	Causal Disentanglement Hidden Markov Model
CSV	Comma-Separated Values
CPS	Cyber-Physical Systems
DANN	Domain-Adversarial Neural Network
DDC	Deep Domain Confusion
DNN	Deep Neural Network
DQN	Deep Q-Network
DSS	Decision Support System
EA	Envelope Analysis
ECU	Electronic Control Unit
EDA	Event-Driven Architecture
FDD	Fault Detection and Diagnosis
FLS	Fuzzy Logic Systems
FL	Federated Learning
GBM	Gradient Boosting Machine
GDPR	General Data Protection Regulation
GRU	Gated Recurrent Unit
GWO	Grey Wolf Optimizer
GWAE	Graph Wavelet Autoencoder
GWVAE	Graph Wavelet Variational Autoencoder
HHT	Hilbert-Huang Transform
ICEEMDAN	Improved Complete Ensemble Empirical Mode Decomposition with Adaptive Noise
IEC	International Electrotechnical Commission
IF	Isolation Forest
IIoT	Industrial Internet of Things
IoT	Internet of Things
IPFS	InterPlanetary File System
ISO	International Organization for Standardization
KSC	K-Shape Clustering
k-NN	k-Nearest Neighbour
LDA	Linear Discriminant Analysis
LOF	Local Outlier Factor
LoRaWAN	Long Range Wide Area Network
LPWAN	Low-Power Wide-Area Network
LSSVM	Least Squares Support Vector Machine
LSTM	Long Short-Term Memory
MARL	Multi-Agent Reinforcement Learning
MAS	Multi-Agent Systems
MCD	Minimum Covariance Determinant
MDP	Markov Decision Process
ML	Machine Learning
MQTT	Message Queuing Telemetry Transport
MSP	Multi-Sensor Platform
M2M	Machine-to-Machine
NB-IoT	Narrowband Internet of Things
NLP	Natural Language Processing
NPPC	Nuclear Power Plants Context
OBD-II	On-Board Diagnostics II
OEA	Overall Equipment Availability
OC-SVM	One-Class Support Vector Machine
OPC UA	Open Platform Communications Unified Architecture
OSVDAE	Optimized Stacked Variational Denoising Autoencoder
PCA	Principal Component Analysis
PB	Prediction-Based
PdM	Predictive Maintenance
PLC	Programmable Logic Controller
PROFINET	Process Field Net
QDA	Quadratic Discriminant Analysis
RF	Random Forest
RL	Reinforcement Learning
RNN	Recurrent Neural Network
RCSR	Remote Control and Service Room
RUL	Remaining Useful Lifetime
SBC	Single-Board Computer
SL	Supervised Learning
SMOTE	Synthetic Minority Over-sampling Technique
SPC	Statistical Process Control
SPHM	Smart Prognostics and Health Management
SPM	Shock Pulse Method
SSL	Secure Sockets Layer
STFT	Short-Time Fourier Transform
SVC	Support Vector Classification
SVR	Support Vector Regression
SVM	Support Vector Machine
TCM	Tool Condition Monitoring
TLS	Transport Layer Security
TNN	Transformer Neural Network
UKF	Unscented Kalman Filtering
VAE	Variational Autoencoder
WNO	Wavelet Neural Operator
