# Supplementary material for: Artificial Intelligence of Things for Next-Generation Predictive Maintenance
Source: Sensors (Basel). 2025 Dec 16;25(24):7636. doi: 10.3390/s25247636 (PMC12737171; doi:10.3390/s25247636)
Supplement: Supplementary file 1 [file sensors-25-07636-s001.zip › sensors-3998014-supplementary.pdf]

PRISMA 2020 Checklist

Manuscript Title: Artificial Intelligence of Things for Next-Generation Predictive Maintenance

Authors: Taimia BITAM, Aya YAHIAOUI, Djallel Eddine BOUBICHE, Rafael Martinez-Peláez, Toral-Cruz Homero, Pablo Velarde-Alvarado

Section & Topic	Item #	PRISMA 2020 Item	Location in Manuscript (Page / Section / Figure/Table)
**TITLE**			
**Title**	1	Identify the report as a systematic review.	Title Page: "Article: Artificial Intelligence of Things for Next-Generation Predictive Maintenance" (A systematic and structured review)
**ABSTRACT**			
**Abstract**	2	See the PRISMA 2020 for Abstracts checklist.	Abstract (Page 1) clearly states it is a "systematic and structured review".
**INTRODUCTION**			
**Rationale**	3	Describe the rationale for the review in the context of existing knowledge.	Section 1. Introduction (Pages 1-2): Context of Industry 5.0, limitations of traditional maintenance, role of AIoT.
**Objectives**	4	Provide an explicit statement of the objective(s) or question(s) the review addresses.	Section 1. Introduction (Page 2, final paragraph) and Section 2.1. Research Questions (Page 3): Explicitly lists RQ1, RQ2, RQ3.
**METHODS**			
**Eligibility criteria**	5	Specify the inclusion and exclusion criteria for the review and how studies were grouped for the syntheses.	Section 2.3. Inclusion and Exclusion Criteria (Pages 3-4). Edge cases are explicitly defined.
**Information sources**	6	Specify all databases, registers, websites, organisations, reference lists and other sources searched or consulted to identify studies. Specify the date when each source was last searched.	Section 2.2. Search Strategy and Data Sources (Page 3): Lists Google Scholar, IEEE Xplore, Scopus, Web of Science. Date of last search: 15 September 2025.
**Search strategy**	7	Present the full search strategies for all databases, registers and websites, including any filters and limits used.	Section 2.2. Search Strategy and Data Sources (Page 3): Provides the full Boolean search query.
**Selection process**	8	Specify the methods used to decide whether a study met the inclusion criteria of the review, including how many reviewers screened each record and each report retrieved, whether they worked independently, and if applicable, details of automation tools used in the process.	Section 2.4. Screening and Selection Process (Page 4): Details independent screening by two reviewers, conflict resolution. Deduplication procedure in Section 2.3.
**Data collection process**	9	Specify the methods used to collect data from reports, including how	Section 2.5. Quality Assessment and Organization (Page 5): Describes assessment based on novelty,

Section & Topic	Item #	PRISMA 2020 Item	Location in Manuscript (Page / Section / Figure/Table)
		many reviewers collected data from each report, whether they worked independently, any processes for obtaining or confirming data from study investigators, and if applicable, details of automation tools used in the process.	rigor, alignment with Industry 5.0. Implied data extraction during review.
**Data items**	10a	List and define all outcomes for which data were sought. Specify whether all results that were compatible with each outcome domain in each study were sought (e.g. for all measures, time points, analyses), and if not, the methods used to decide which results to collect.	Section 2.1. Research Questions (Page 3) defines the scope. Data extracted (AI techniques, IIoT components, sector, performance) is evident in the synthesis tables (e.g., Tables 6-24).
	10b	List and define all other variables for which data were sought (e.g. participant and intervention characteristics, funding sources). Describe any assumptions made about any missing or unclear information.	Variables extracted include: publication year, AI method, IIoT architecture, application sector, Industry 5.0 value. Implied from the structure of the review.
**Study risk of bias assessment**	11	Specify the methods used to assess risk of bias in the included studies, including details of the tool(s) used, how many reviewers assessed each study and whether they worked independently, and if applicable, details of automation tools used in the process.	Section 2.5. Quality Assessment and Organization (Page 5): Assessment based on "methodological clarity, technical rigor, and alignment with the Industry 5.0 paradigm".
**Effect measures**	12	Specify for each outcome the effect measure(s) (e.g. risk ratio, mean difference) used in the synthesis or presentation of results.	Not applicable for this methodology-focused review. Outcomes are qualitative (techniques, architectures, challenges) and quantitative performance metrics (accuracy, RMSE, etc.) as reported in primary studies.
**Synthesis methods**	13a	Describe the processes used to decide which studies were eligible for each synthesis (e.g. tabulating the study intervention characteristics and comparing against the planned groups for each synthesis (item #5)).	Section 2.4 & 2.5: The 153 studies were thematically synthesized into dimensions (AI applications, IIoT applications, AIoT methodologies) as outlined in Section 5.
	13b	Describe any methods required to prepare the data for presentation or synthesis, such as handling of missing summary statistics, or data conversions.	Data was synthesized narratively and in comparative tables. Performance metrics are reported as presented in the primary studies.

Section & Topic	Item #	PRISMA 2020 Item	Location in Manuscript (Page / Section / Figure/Table)
	13c	Describe any methods used to visually present results of the synthesis (e.g. tables, flow diagrams).	Results are presented in structured tables (Tables 6-24) and a flow diagram (Figure 1). Section 5 is organized thematically.
	13d	Describe any methods used to synthesize results and provide a rationale for the choice(s). If meta-analysis was performed, describe the model(s), method(s) to identify the presence and extent of statistical heterogeneity, and software package(s) used.	A narrative synthesis was performed, grouping studies by methodology (AI technique, IIoT application, AIoT paradigm). Rationale is the interdisciplinary and heterogeneous nature of the field.
	13e	Describe any methods used to explore possible causes of heterogeneity among study results (e.g. subgroup analysis, meta-regression).	Heterogeneity is explored thematically in the Discussion (Section 6), analyzing differences across sectors, techniques, and architectures.
	13f	Describe any sensitivity analyses conducted to assess the robustness of the synthesized results.	Not applicable. This is a qualitative, scoping synthesis, not a quantitative meta-analysis.
**Reporting bias assessment**	14	Describe any methods used to assess risk of bias due to missing results in a synthesis (arising from reporting biases).	Not applicable for this type of review. The focus is on mapping the field, not estimating an effect size.
**Certainty assessment**	15	Describe any methods used to assess certainty (or confidence) in the body of evidence for an outcome.	Not applicable. This review does not assess the certainty of evidence for a specific intervention.
**RESULTS**			
**Study selection**	16a	Describe the results of the search and selection process, from the number of records identified in the search to the number of studies included in the review, ideally using a flow diagram.	Section 2.4. Screening and Selection Process (Page 4) and Figure 1 (PRISMA Flow Diagram).
	16b	Cite studies that might appear to meet the inclusion criteria, but which were excluded, and explain why they were excluded.	The exclusion reasons with counts are provided in Section 2.4 and detailed in Figure 1.
**Study characteristics**	17	Cite each included study and present its characteristics.	The 153 included studies are cited throughout Section 5 and their characteristics (AI technique, focus, sector) are synthesized in Tables 6-24. The full list is in Supplementary File S1.
**Risk of bias in studies**	18	Present assessments of risk of bias for each included study.	Not formally presented per study, but the limitations of individual studies/approaches are discussed in the "Limitations" columns of synthesis tables (Tables 6-24) and in Section 6.2.
**Results of individual studies**	19	For all outcomes, present, for each study: (a) summary statistics for each group (where applicable) and (b) an	For the methodological focus of this review, key contributions and performance metrics (accuracy, RMSE, etc.) from individual studies are presented

Section & Topic	Item #	PRISMA 2020 Item	Location in Manuscript (Page / Section / Figure/Table)
		effect estimate and its precision (e.g. confidence/credible interval), ideally using structured tables or plots.	in the "Key Contributions" and "Performance Metrics" columns of **Tables 6-24**.
**Results of syntheses**	20a	For each synthesis, briefly summarise the characteristics and risk of bias among contributing studies.	The characteristics of studies in each synthesis (e.g., AI for FDD, IIoT in Manufacturing) are summarized in the introductory text of each subsection in Section 5 and in the respective tables.
	20b	Present results of all statistical syntheses conducted. If meta-analysis was done, present for each the summary estimate and its precision (e.g. confidence/credible interval) and measures of statistical heterogeneity. If comparing groups, describe the direction of the effect.	Not applicable. No statistical meta-analysis was performed.
	20c	Present results of all investigations of possible causes of heterogeneity among study results.	Thematic causes of heterogeneity (e.g., dataset quality, sector specificity) are discussed in Section 6.2 (Limitations and Gaps).
	20d	Present results of all sensitivity analyses conducted to assess the robustness of the synthesized results.	Not applicable.
**Reporting biases**	21	Present assessments of risk of bias due to missing results (arising from reporting biases) for each synthesis assessed.	Not applicable.
**Certainty of evidence**	22	Present assessments of certainty (or confidence) in the body of evidence for each outcome assessed.	Not applicable.
**DISCUSSION**			
**Discussion**	23a	Provide a general interpretation of the results in the context of other evidence.	Section 6. Discussion (Pages 52-54) interprets the findings, linking them to the Industry 5.0 paradigm and prior knowledge.
	23b	Discuss any limitations of the evidence included in the review.	Section 6.2. Limitations and Gaps in Current Research (Page 53) details limitations of the reviewed studies and the field.
	23c	Discuss any limitations of the review processes used.	Section 6.2. also acknowledges review process limitations (e.g., model complexity, data quality issues in primary studies).
	23d	Discuss implications of the results for practice, policy, and future research.	Section 6.3. Future Perspectives and Research Opportunities (Pages 53-54) details implications for future research. Section 7. Conclusions summarizes practical implications.
**OTHER INFORMATION**			
**Registration and protocol**	24a	Provide registration information for the review, including register name	The review was not registered.

Section & Topic	Item #	PRISMA 2020 Item	Location in Manuscript (Page / Section / Figure/Table)
		and registration number, or state that the review was not registered.	
	24b	Indicate where the review protocol can be accessed, or state that a protocol was not prepared.	A protocol was not prepared.
	24c	Describe and explain any amendments to information provided at registration or in the protocol.	Not applicable.
**Support**	25	Describe sources of financial or non-financial support for the review, and the role of the funders or sponsors in the review.	Support information would be on the title page (not provided in the excerpt).
**Competing interests**	26	Declare any competing interests of review authors.	Competing interests declaration would be on the title page (not provided in the excerpt).
**Availability of data, code and other materials**	27	Report which of the following are publicly available and where they can be found: template data collection forms; data extracted from included studies; data used for all analyses; analytic code; any other materials used in the review.	Supplementary File S1 (Inclusion_List_153_Studies.csv) provides the list of analyzed studies. The search strategy is fully reported in Section 2.2.
